# Logically Inferred Tuberculosis Transmission (LITT): A Data Integration Algorithm to Rank Potential Source Cases

**DOI:** 10.3389/fpubh.2021.667337

**Published:** 2021-06-21

**Authors:** Kathryn Winglee, Clinton J. McDaniel, Lauren Linde, Steve Kammerer, Martin Cilnis, Kala M. Raz, Wendy Noboa, Jillian Knorr, Lauren Cowan, Sue Reynolds, James Posey, Jeanne Sullivan Meissner, Shameer Poonja, Tambi Shaw, Sarah Talarico, Benjamin J. Silk

**Affiliations:** ^1^Division of Tuberculosis Elimination, Centers for Disease Control and Prevention, Atlanta, GA, United States; ^2^TB Control Branch, California Department of Public Health, Richmond, CA, United States; ^3^Los Angeles County Department of Public Health, Los Angeles, CA, United States; ^4^New York City Department of Health and Mental Hygiene, Queens, NY, United States

**Keywords:** tuberculosis, whole-genome sequencing, transmission, genomic epidemiology, cluster investigation

## Abstract

Understanding tuberculosis (TB) transmission chains can help public health staff target their resources to prevent further transmission, but currently there are few tools to automate this process. We have developed the Logically Inferred Tuberculosis Transmission (LITT) algorithm to systematize the integration and analysis of whole-genome sequencing, clinical, and epidemiological data. Based on the work typically performed by hand during a cluster investigation, LITT identifies and ranks potential source cases for each case in a TB cluster. We evaluated LITT using a diverse dataset of 534 cases in 56 clusters (size range: 2–69 cases), which were investigated locally in three different U.S. jurisdictions. Investigators and LITT agreed on the most likely source case for 145 (80%) of 181 cases. By reviewing discrepancies, we found that many of the remaining differences resulted from errors in the dataset used for the LITT algorithm. In addition, we developed a graphical user interface, user's manual, and training resources to improve LITT accessibility for frontline staff. While LITT cannot replace thorough field investigation, the algorithm can help investigators systematically analyze and interpret complex data over the course of a TB cluster investigation.

**Code available at:** https://github.com/CDCgov/TB_molecular_epidemiology/tree/1.0; https://zenodo.org/badge/latestdoi/166261171.

## Introduction

Public health investigations now routinely use phylogenetic analysis of whole-genome sequencing (WGS) data to help characterize infectious disease transmission (1, 2). By identifying chains of recent *Mycobacterium tuberculosis* (*Mtb*) transmission, health officials can detect and treat additional cases of tuberculosis (TB) disease, identify contacts with latent TB infection for preventive therapy, and target populations and settings where transmission may have occurred for additional public health interventions. Thus, identifying potential source cases can help public health staff to determine the best way to use limited resources to prevent further transmission. However, cases in the same chain of transmission often have *Mtb* strains that are genetically indistinguishable or very similar (3). Therefore, WGS data must be considered within clinical and epidemiologic contexts to reliably infer TB sources and directionality of transmission. This is generally done manually by frontline investigators, which can be time consuming and inefficient.

Several algorithms have been published to infer transmission chains from WGS and epidemiologic data, including SeqTrack (4), outbreaker (5), Structured Coalescent Transmission Tree Inference (SCOTTI) (6), TransPhylo (7), and phybreak (8). Although their flexible designs allow for diverse applications across many pathogens, these algorithms rely heavily on complete sequencing data and genomic diversity among isolates. This reliance makes them less applicable to *Mtb*, which can have relatively low mutation rates (9) and less genomic diversity, while excluding cases that do not have sequence data. These algorithms also generally rely on sample collection dates that are not necessarily representative of transmission timing in the context of TB because TB patients can have long periods of latent infection or undiagnosed TB disease. In addition, cases in young children (<10 years old) and cases with exclusively extrapulmonary sites of disease are typically not considered contagious (10), and should not be designated as source cases, but these algorithms have no methods to treat certain cases as unlikely to transmit. Furthermore, they cannot make predictions for cases that do not have sequencing data. For example, over 20% of cases in the United States do not have WGS available because they are culture-negative (11).

We developed the Logically Inferred Tuberculosis Transmission (LITT) algorithm, which is based on the logic and methods applied by expert U.S. state and local TB control program staff in the field, to evaluate evidence and make inferences during investigations of *Mtb* transmission events. Once data are compiled, LITT runs quickly (<60 seconds on a laptop analyzing a large dataset). Here we describe how LITT integrates WGS, clinical, and epidemiologic data to identify and rank potential source cases (from most to least likely to be the source) for cluster investigations. We evaluated LITT using retrospective data from diverse transmission circumstances in multiple high-burden jurisdictions in the United States. We then reviewed discrepancies when LITT predictions for source cases did not match presumed source cases identified by TB investigations.

## Methods

### Data Compilation

We used available molecular and epidemiologic data from 56 clusters that were investigated between 2011 and 2019 by the California Department of Public Health (CDPH), the Los Angeles County Department of Public Health (LA DPH), or the New York City Department of Health and Mental Hygiene (NYC DOHMH). These clusters ranged from two to 69 cases, represented a range of transmission scenarios, and were split into a training set (30 clusters) to develop the algorithm and a test set (26 clusters) to assess performance. Clusters were defined by the investigators and included cases with matching or similar genotype results or an epidemiologic relationship to a genotyped case if no genotype was available. The presumed source case was defined as the most likely source identified by the investigation. Using standardized definitions defined by local investigators, we recorded epidemiologic links between patients based on close associations or shared locations. The strength of these epidemiologic links (i.e., definite, probable, or possible based on pre-established definitions) were also recorded (Supplementary Table 1). We summarized WGS results in a single nucleotide polymorphism (SNP) matrix, which indicates the number of base-pair differences between each pair of isolates in the cluster. See Appendix 1.1 in Supplementary Material for additional details.

For adult patients with pulmonary or laryngeal TB, we defined infectious period (IP) start dates as the IP start date recorded in the investigation records, if available; otherwise, we used 3 months before the earliest date that we could determine the patient had TB (using available surveillance and investigation data), which is based on U.S. Centers for Disease Control and Prevention (CDC) guidelines (10). We used the IP end date from the investigation records, if the local program had calculated this date. Otherwise, we calculated an IP end date as 2 weeks after treatment start date, if available. Because patients with exclusively extrapulmonary TB (not including laryngeal TB) and pediatric TB cases typically are not infectious, LITT also calculates an infection acquisition (IA) period to define a time window when a given case could have been infected, such as date of birth or date of arrival in the United States for persons born outside of the United States. See Appendix 1.2 in Supplementary Material for more details on date calculations. CDC and the involved public health departments determined that this project did not constitute human subjects research and did not require Institutional Review Board approval.

### LITT Algorithm

**Figure 1:**
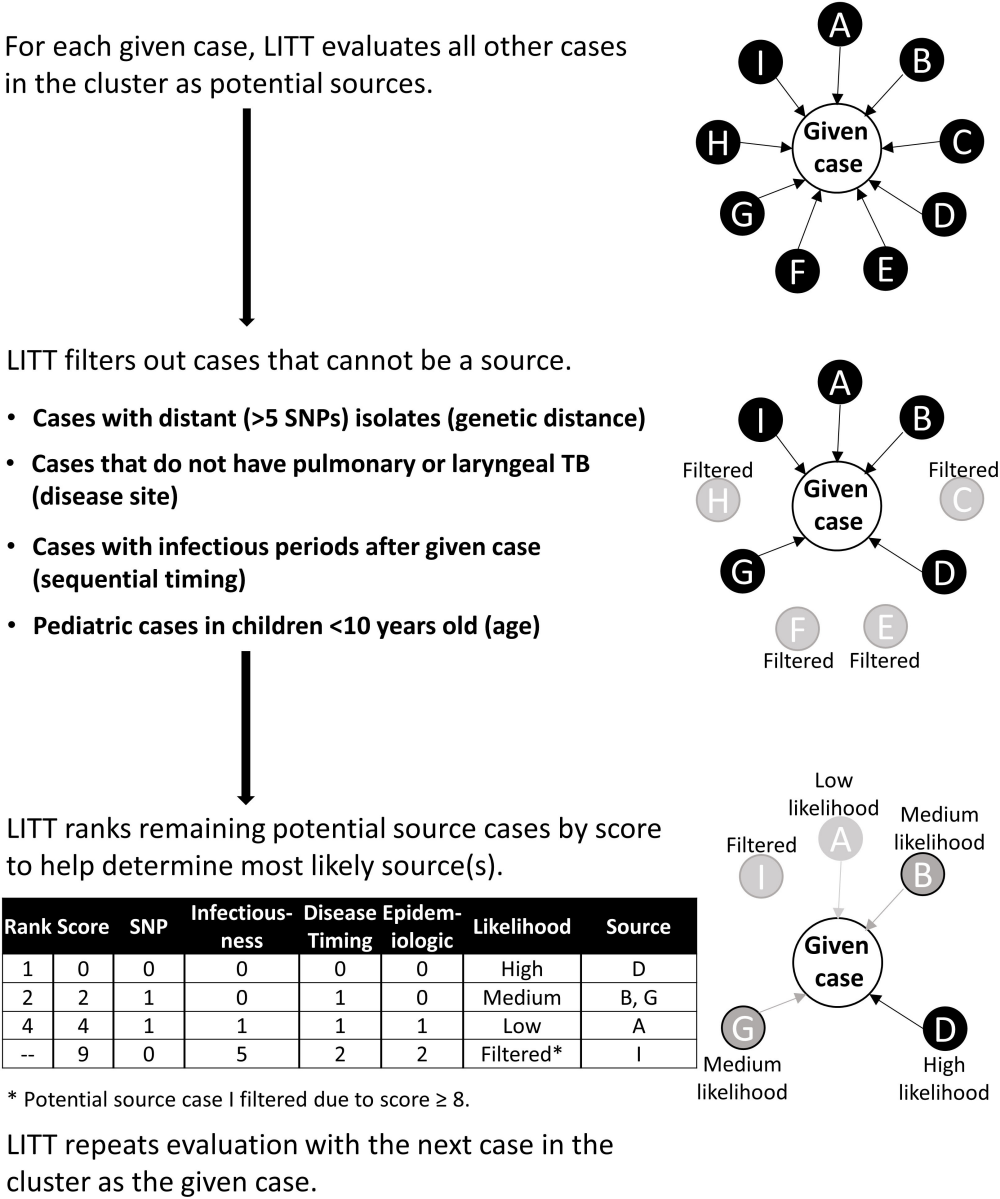
Schematic overview of Logically Inferred Tuberculosis Transmission (LITT) algorithm. LITT picks one case to be the given case then looks at all other possible source cases in the cluster (cases A–I). First, LITT filters out cases that could not be a source (cases C, E, F, and H) based on genetic distance between isolates (i.e., distance in single nucleotide polymorphisms, or SNPs), a non-infectious disease site (i.e., extrapulmonary disease), sequential timing (i.e., cases with infectious periods after given case), and young age (i.e., typically non-infectious). Next, LITT scores the remaining cases and ranks these cases as potential sources by numeric score (Table 1, Appendix 1 in Supplementary Material). LITT then repeats this process for each given case in the cluster.

For each given case, LITT evaluates all other cases in a cluster as potential source cases and outputs a filtered, ranked list of potential source cases (Figure 1). Briefly, LITT first filters out any case that does not meet the following four criteria (i.e., could not logically be the source case):

1. Genetic distance: have an *Mtb* isolate within 5 SNPs of the given case's isolate, or, if no SNP distance is available, have an epidemiologic link or shared risk factor (e.g., homelessness),
2. Disease site: have an infectious form of TB disease,
3. Sequential timing: be infectious before the earliest known time a given case had TB disease and after the IA start, and
4. Age: be a patient with pulmonary capacity capable of transmitting *Mtb*, defined as a patient who is at least 10 years old at the time of TB diagnosis (10).

For any remaining potential sources, LITT assigns each source case a score based on four criteria (Table 1):

1. SNP rating: the number of SNPs between isolates for the source and given case,
2. Infectiousness rating: source case's degree of infectiousness based on the presence or absence of acid-fast bacilli noted on sputum microscopy or cavitation noted on a chest radiograph,
3. Disease timing rating: timing of cases' infectious periods, and
4. Epidemiologic and shared risk factor rating: epidemiologic relationships between source and given case (strength of link), or shared risk factors (e.g., homelessness) that are relevant to the cluster.

**Table 1:** Logically Inferred Tuberculosis Transmission (LITT) scoring system used to rank potential tuberculosis (TB) source cases^*^.

**Criteria**	**Score**	**Relationship between given and potential source case characteristics**
SNP rating	0	0 SNP difference between *Mycobacterium tuberculosis* isolates
	1	1 SNP difference between *M. tuberculosis* isolates
	2	2 SNP difference between *M. tuberculosis* isolates
	3	3 SNP difference between *M. tuberculosis* isolates
	4	4 SNP difference between *M. tuberculosis* isolates
	5	5 SNP difference between *M. tuberculosis* isolates
	Missing	No SNP data available
Infectiousness rating	0	Source case has cavitary disease on chest radiograph regardless of sputum smear microscopy results
	1	Source case has no evidence of cavitary disease on chest radiograph, but positive sputum smear microscopy
	5	Source case has no evidence of cavitary disease on chest radiograph, and negative sputum smear microscopy
Disease timing rating	0	Source IP start date is before given case IP start date and source IP end <2 years before given case IP start date
	1	Source IP end date ≥2 years before given case IP start date
	2	Source IP start date after given case IP start date (but passes sequential timing filter)
Epidemiologic and shared risk factor rating	0	Definite epidemiologic link
	1	Probable epidemiologic link
	2	Possible epidemiologic link
	2–3	No epidemiologic link but shared risk factor (e.g., shelter, substance use)
	3	No epidemiologic link or shared risk factor

* *For each potential source of a given TB case, LITT assigns a value for the SNP rating, infectiousness rating, disease timing rating, and epidemiologic and shared risk factor rating. These ratings are summed to create the score (total score uses all four; without-SNP score uses all but the SNP rating), which is used to rank the potential sources. Note that lower scores indicate a higher likelihood of being a potential source for each given case. See Appendix 1.4 in Supplementary Material for more details on scoring*.

*SNP, single nucleotide polymorphism; IP, infectious period*.

Appendix 1 in Supplementary Material describes LITT filtering (Appendix 1.3) and scoring (Appendix 1.4), with an example (Appendix 1.5), in more detail. LITT sums the four ratings for each potential source case to calculate a total score. The score is designed *inversely* so that the lower the value, the more likely that case is the presumed source from the investigation for the given case. A total score cutoff ≥8 is used to filter out unlikely potential source cases (based on analysis of the investigation presumed sources; Appendix 1.6 in Supplementary Material). The remaining potential sources are ranked from most to least likely for the given case (i.e., lowest to highest total score), and categorized as high, medium, or low likelihood of being the source, based on an analysis of the investigation presumed source scores (Appendix 1.4 in Supplementary Material). If no SNP distance is available for the given case-potential source pair (i.e., one or both do not have WGS data), a “without-SNP” score is calculated and used to rank the potential source. LITT then repeats the algorithm with the next case in the cluster being defined as the given case. This procedure is repeated for all remaining cases in the cluster. Note that LITT can evaluate clusters where some cases have WGS data while some (or all) do not.

### LITT Evaluation and Detailed Review

We started with a comparative evaluation of previously published algorithms (4–8) and LITT, as applied to the largest cluster in the dataset (details in Appendix 2 text in Supplementary Material). To further evaluate LITT, we then applied LITT to the full dataset from the cluster investigations (see Data Compilation above). We used the presumed source cases identified by investigators as our gold standard in comparison with the most likely source case identified by LITT or the other algorithms (see Supplementary Figure 1B for distribution of presumed source determinations among clustered cases). If the presumed source from the investigation was ranked first by LITT, we considered the algorithm in agreement with investigators, even if LITT identified another potential source that was tied for first. Likewise, for given cases with multiple presumed sources identified by investigators due to uncertainty during the investigation, as long as one of the presumed sources was ranked first by LITT, we also considered this as an agreement.

We also performed a detailed review for cases in the three clusters investigated by CDPH and local partners. Detailed reviews were conducted for any given case where: (a) LITT identified a different most likely source case from the investigation records, (b) LITT and the investigation agreed on a most likely source, however, LITT found an additional case that was equally qualified to be the most likely source case, (c) the investigation identified multiple presumed source cases but LITT ranked one above the other, (d) the investigation identified a source case but LITT did not, or (e) the investigation did not identify a source case but LITT did. For each scenario, we reviewed all available investigation data and classified each difference as confirming or refuting the LITT prediction, or as lacking sufficient data for classification.

### Code Availability

A LITT user's manual, training (mock) datasets, training presentation, input file templates, and all code written in R (12) are available at: https://github.com/CDCgov/TB_molecular_epidemiology. In addition, CDC's Office of Advanced Molecular Detection (OAMD) has LITT running as an R Shiny application (Supplementary Figure 2) on the OAMD Portal, which is available at: https://amdportal-sams.cdc.gov/portal/. Access requests can be sent to TBGenotyping@cdc.gov.

## Results

### LITT Evaluation

In the comparative evaluation of LITT and previously published algorithms (4–8), we ran each algorithm on our largest cluster three times with the same settings (to assess reproducibility of the findings given that several algorithms use random sampling from a posterior distribution) and compared the results with the presumed sources from the investigation. We found that LITT identified the same most likely source case in each of three evaluation runs and identified the same most likely source as investigators for 17 (89.5%) of 19 cases in a large cluster (*n* = 69 total cluster cases). This finding contrasted significantly with evaluation findings for the next best algorithm, SCOTTI, which identified the same most likely source as investigators for an average of 3.3 (17.5%) cases but also identified different sources for two cases in different evaluation runs with the same settings (Supplementary Table 2).

**Figure 2:**
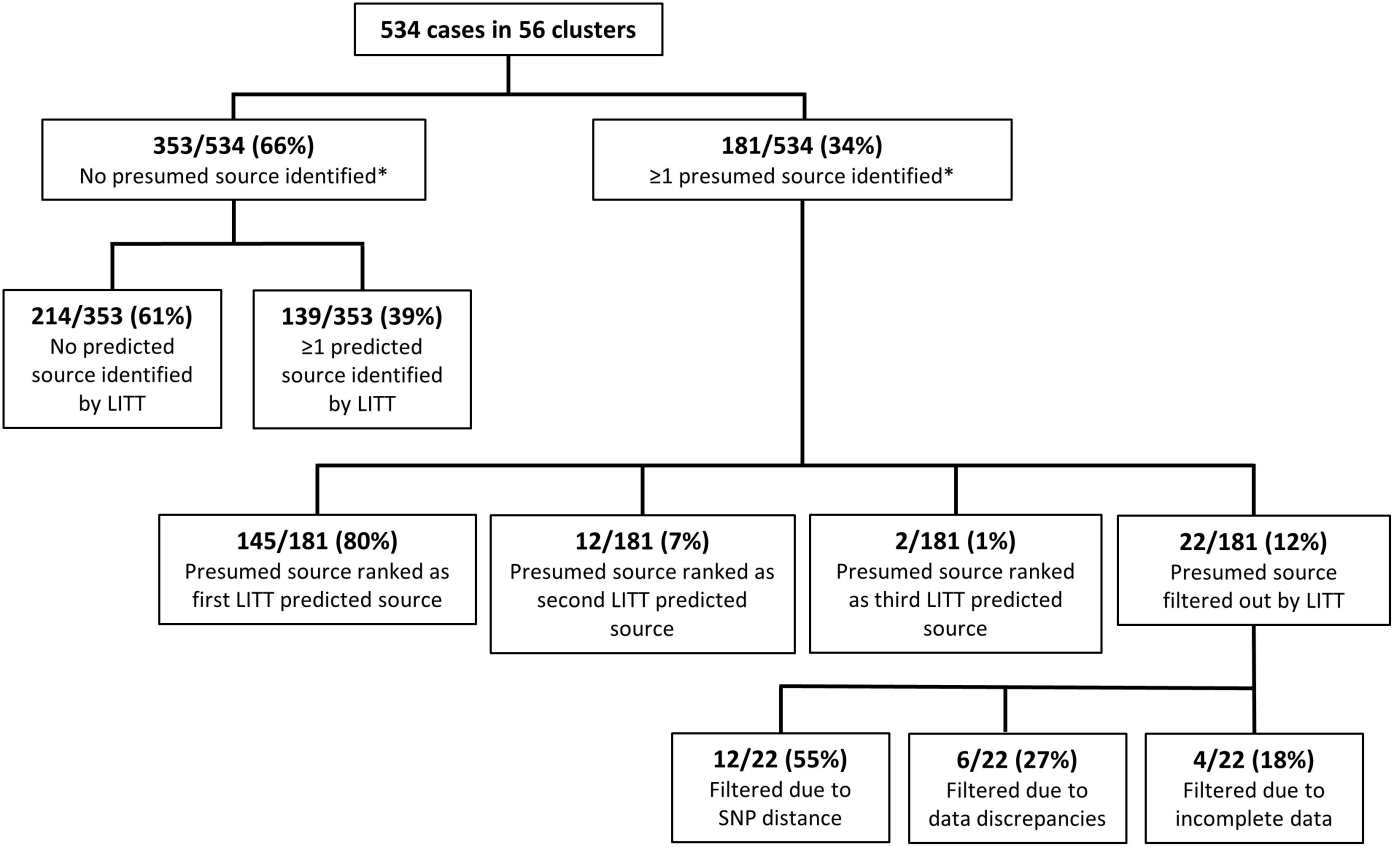
Comparison of the Logically Inferred Tuberculosis Transmission (LITT) algorithm's predicted source cases to presumed sources identified by local investigators for all 56 TB clusters. Shown here are all cases in all clusters. Supplementary Figure 3 splits the results by whether the cluster was in the training or test set. *Presumed source cases of *Mycobacterium tuberculosis* transmission identified by public health investigations conducted by the California Department of Public Health, Los Angeles County Department of Public Health, New York City Department of Health and Mental Hygiene, and their partners.

We then applied LITT to 56 clusters composed of 534 cases (cluster size range: 2–69 cases, Supplementary Figure 1), of which 181 (34%) cases had at least one presumed source case identified by local investigators (Figure 2, Supplementary Figure 3). When at least one presumed source case was identified, the given case of interest was more likely to be U.S.-born (61%) and, by design, have at least one epidemiologic link identified (99%) compared to cases that did not have a presumed source identified (38 and 45%, respectively) (Table 2). All pediatric cases had a presumed source identified by local investigators, reflecting the source case investigations commonly performed for pediatric cases, while as cases got older, the proportion with a presumed source case decreased. WGS was available for all clusters, but 32 (57%) of 56 clusters were missing WGS data on at least one case.

**Table 2:** Select demographic and clinical characteristics of 534 tuberculosis cases in 56 clusters – California, Los Angeles County, and New York City, 2011–2019.

**Characteristic**	Given cases with no presumed source (*n* = 353)^*^	Given cases with at least one presumed source (*n* = 181)^*^
**Age group (years)**		
0–9	0 (0%)	29 (16%)
10–24	52 (15%)	43 (24%)
25–44	115 (33%)	63 (35%)
45–64	137 (39%)	30 (17%)
65+	49 (14%)	16 (9%)
**Sex**		
Female	108 (31%)	71 (39%)
Male	245 (69%)	110 (61%)
**Place of birth**		
United States	135 (38%)	110 (61%)
Other country	217 (61%)	71 (39%)
Unknown	1 (0%)	0 (0%)
**Race/ethnicity**		
Hispanic	166 (47%)	87 (48%)
Non-Hispanic Asian	81 (23%)	38 (21%)
Non-Hispanic Black	80 (23%)	35 (19%)
Non-Hispanic White	18 (5%)	20 (11%)
Multiple/Other	4 (1%)	1 (1%)
Unknown	4 (1%)	0 (0%)
**Sputum smear microscopy**		
Positive	229 (65%)	86 (48%)
Negative	111 (31%)	63 (35%)
Unknown	13 (4%)	32 (18%)
**Evidence of cavitary disease on chest radiograph**		
Cavitary	91 (26%)	39 (22%)
Non-Cavitary	231 (65%)	117 (65%)
Unknown	31 (9%)	25 (14%)
**HIV Status**		
Positive	29 (8%)	1 (1%)
Negative	252 (71%)	150 (83%)
Unknown	72 (20%)	30 (17%)
Whole-genome sequencing (WGS) data available	292 (83%)	144 (80%)
Symptom onset or infectious period start data ascertained from investigation	272 (77%)	118 (65%)
Has at least one epidemiologic link^**^	158 (45%)	179 (99%)

* *Having a presumed source indicates that the local investigation identified a most likely presumed source for a given case. Investigations were conducted by the California Department of Public Health, Los Angeles County Department of Public Health, New York City Department of Health and Mental Hygiene, and their partners*.

** *In total there were 490 epidemiologic links identified (233 definite, 93 probable, and 164 possible epidemiologic links)*.

Of the 181 cases with a presumed source case identified by the investigation, LITT ranked the presumed source first for 145 (80.1%) cases (Figure 2). An additional 14 (7.7%) of the investigation presumed sources were ranked second or third by LITT (i.e., LITT agreed with the investigation that these cases could be a source but identified other more likely potential source cases). The remaining 22 cases had their presumed source filtered out by LITT. Twelve (55%) of these 22 filtered presumed source cases had large SNP distances (median 7; range 6–59 SNPs). The investigation generally identified these cases as the most likely source before WGS results were available. Another six cases were filtered because of data discrepancies, such as inconsistent date or site of disease. The remaining four filtered cases were not included in the analysis because data were incomplete (e.g., missing timing of disease). In addition, LITT was able to identify at least one potential source case for 139 of the 353 cases that had no presumed source case identified by local investigators. The remaining 214 cases had no source identified by either the investigators or LITT. These cases were included in the investigation because of their genotype, but were then excluded by WGS results (SNP distance >5 from any other case in the cluster), or had no WGS and no epidemiologic links to tie them to another case in the cluster. Thus, taken together, there was high concordance (80%) between the LITT predictions and the investigation presumed source cases when sufficient data were available.

**Figure 3:**
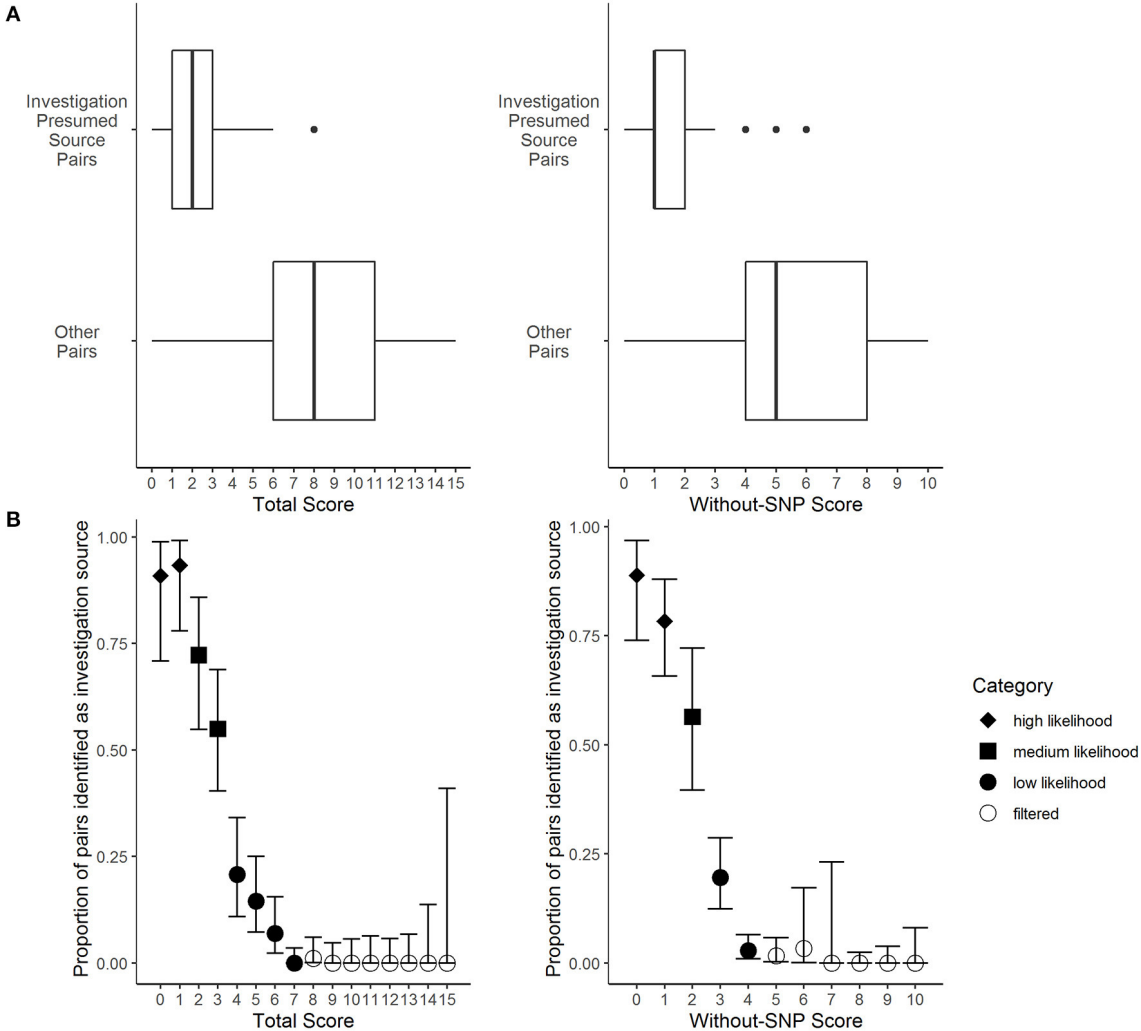
Frequency distributions of the Logically Inferred Tuberculosis Transmission (LITT) algorithm's scoring for TB given case-potential source pairs by local investigation presumed source determinations. Presumed source cases of *Mycobacterium tuberculosis* transmission identified by public health investigations conducted by the California Department of Public Health, Los Angeles County Department of Public Health, New York City Department of Health and Mental Hygiene, and their partners. Data were subset from all 873 potential sources that passed LITT's filters for 177 given cases that had an investigation presumed source in the data set (presumed sources for four given cases had insufficient data). See Table 1 for LITT scoring system. **(A)** The LITT total scores (left) or without-SNP scores (right) by case pair status, where “investigation presumed source pairs” indicate presumed sources identified by the investigation and “other pairs” indicate all other potential sources that passed LITT's filters (Wilcoxon rank sum tests, *p*-values < 0.0001). **(B)** The LITT total score (left) or without-SNP score (right), where points indicate the proportions of pairs with that score that were presumed source pairs identified by local investigation. Bars indicate 95% confidence intervals calculated assuming point estimates were binomially distributed. Point shapes indicate the likelihood category assigned to each score (i.e., high, medium, or low likelihood, or filtered).

The presumed sources identified by investigators had significantly lower total LITT scores (median total LITT score: 2) compared to other potential sources identified by LITT (median total LITT score: 8) (Wilcoxon rank sum test, *p*-value: < 0.0001) (Figure 3A). Lower LITT scores indicate the potential source is more likely to be source for the given case. Likewise, the investigation presumed sources also had significantly lower without-SNP LITT scores (median without-SNP LITT score: 1) compared with other potential sources identified by LITT (median without-SNP LITT score: 5) (*p*-value: < 0.0001) (Figure 3A). Furthermore, potential sources with lower scores were more likely to be identified by investigators as the most likely source (Figure 3B). Based on these results, we categorized total score and without-SNP score values into high, medium, and low likelihood to help LITT users prioritize potential sources for investigation and public health action (Appendix 1.6 in Supplementary Material).

### Detailed Review

**Figure 4:**
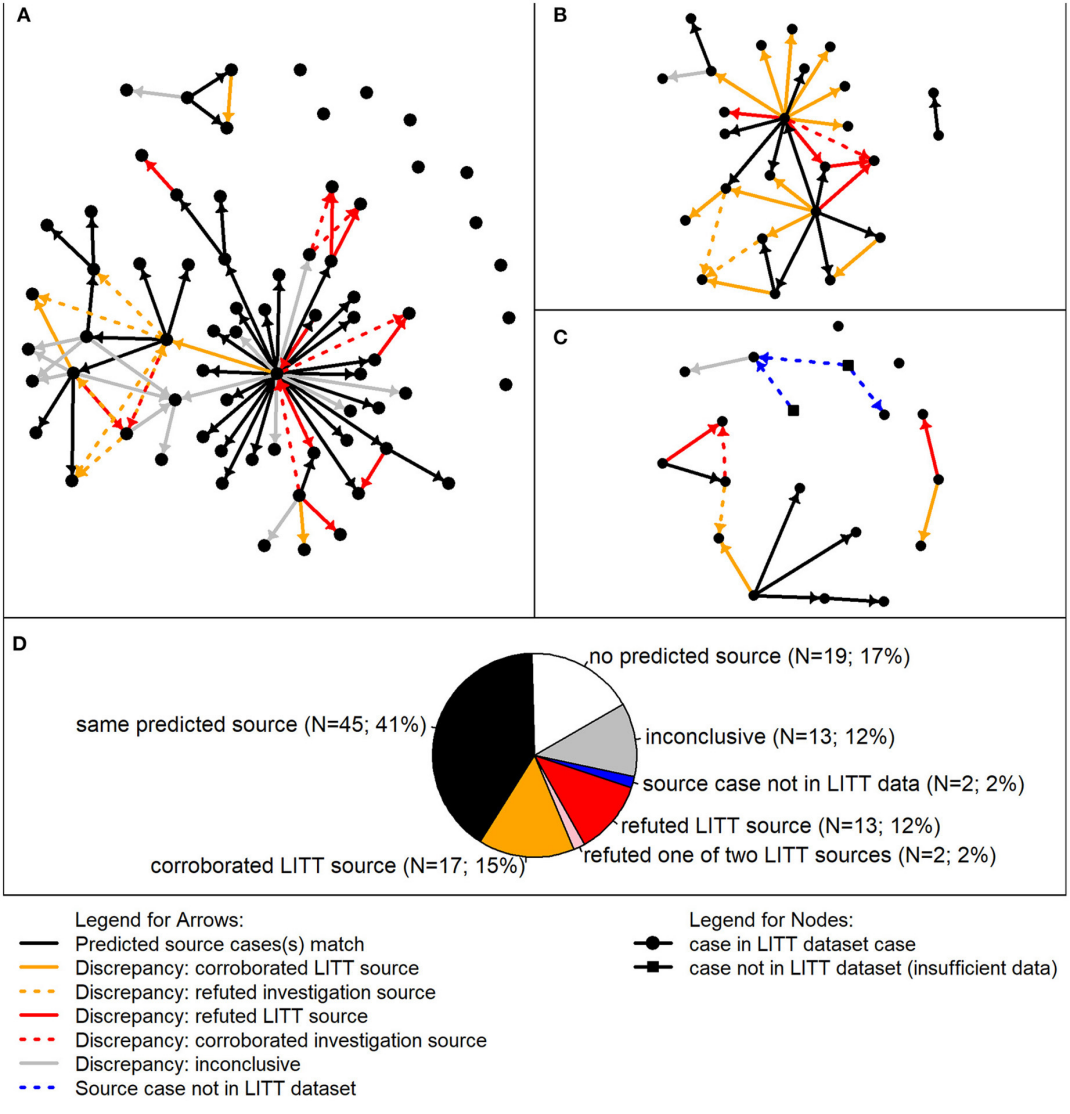
Detailed review of most likely TB source cases predicted by the Logically Inferred Tuberculosis Transmission (LITT) algorithm vs. presumed sources identified by local investigation. In the analysis of discrepancies, 111 given cases associated with three clusters were included. Presumed source cases of *Mycobacterium tuberculosis* transmission were identified by public health investigations conducted by the California Department of Public Health and their partners. **(A–C)** Transmission networks for three TB clusters. Each circle represents a case in the LITT dataset; squares indicate cases not in the LITT dataset (i.e., insufficient data on disease timing and clinical characteristics). Arrows point from a source case identified by LITT and/or the investigation to a given case. Solid arrows point from the most likely source predicted by LITT to the given case. Black solid arrows indicate that the source case was also determined to be the most likely by the investigation. Dashed arrows indicate the source case was determined to be the most likely by the investigation, but not by LITT. A detailed review was performed when the most likely source case predicted by LITT was different than the presumed source case determined by the investigation (i.e., all arrows except the black arrows). The arrows are colored by detailed review results. Gold: review corroborated LITT or refuted the investigation in favor of LITT; red: review refuted the most likely LITT source or corroborated the investigation source over LITT; and gray: inconclusive detailed review (i.e., insufficient data to corroborate or refute). Blue dashed lines indicate possible connections from cases not in the LITT dataset. **(D)** Aggregate summary of detailed review findings combining cases from the three clusters. Relative numbers of given cases in each category are included by the same color code (see legend). In other words, the color of the arrows (solid or dashed in **(A–C)** going in to the given case corresponds where that given case was counted in the pie chart, with the exception of the pink wedge, which corresponds to given cases having two LITT potential sources, one of which was refuted (red) and the other was identified by the investigation (black).

We were interested in better understanding the differences between the LITT and investigation results, so we performed a detailed review of all 111 cases associated with the three clusters investigated by CDPH (Figure 4). Of these cases, LITT and the local investigators agreed on the most likely source case for 45 (41%) cases. Nineteen (17%) had no source case identified by either LITT or investigators, and 13 (12%) had insufficient data on the presumed source case identified by investigators to corroborate or refute the most likely source identified by LITT. The detailed review focused on the remaining 34 (31%) cases.

Of the remaining 34 cases with a different source case identified and sufficient data for review, two (6%) cases had an investigation presumed source who resided outside the United States. These presumed source cases were not eligible for consideration by LITT because of lack of additional data, such as the timing of when they were infectious, site of disease, and infectiousness (Figure 4C). The detailed review also refuted LITT's most likely sources for 13 (38%) and corroborated LITT's sources for 17 (50%) of 34 cases. Another two cases each had two sources identified by LITT, one that was corroborated and one that was refuted by the review.

Among the 13 source cases identified by LITT that were refuted upon further review, six were the result of data entry errors (missing or improperly labeled epidemiologic links, or incorrect dates). Three additional LITT-identified source cases were refuted because of tuberculin skin test (TST) history. The three given cases associated with these sources had documented TST conversions from negative to positive before LITT's most likely source was infectious. Three additional case reviews refuted LITT because of patient characteristics that were not considered strong enough to be an epidemiologic link for inclusion in the LITT dataset (e.g., two cases in persons who lived in the same small neighborhood). However, these three presumed source cases were listed as potential sources and ranked second by LITT. One other case was refuted because the given case occurred in a person who first arrived in the United States after the infectious period of LITT's most likely source. After this review, we added the infection acquisition start to the LITT algorithm (Appendix 1.2 in Supplementary Material). In addition to these 13 cases, two cases each had two most likely source cases identified by LITT; the review corroborated one of the source cases while refuting the other based on TST history.

Of the 17 cases where the detailed review corroborated LITT, seven initially had no source identified by the local investigation, but the detailed review agreed with LITT's most likely source case. The 10 other cases had an initial source assessment made, but the presumed source identified had not been updated as new data became available that contradicted this designation, such as WGS results or updated infectious periods.

## Discussion

The field of molecular epidemiology for investigating recent TB transmission is rapidly evolving, with WGS increasingly being used in public health practice to detect clusters and identify and characterize recent transmission (13–16). However, analysis and use of WGS, such as SNP cutoffs, is still not standardized (17, 18). WGS can be used to rule out transmission, but cannot be used alone to determine if direct transmission occurred, especially when there is low genetic diversity (19, 20). In addition, it cannot be used to infer direction of transmission without additional data. Fully understanding TB transmission requires not just an analysis of *Mtb* sequences, but also integration of the clinical and epidemiological characteristics of the patients (21, 22).

We initially conducted a literature review to identify candidate algorithms that infer transmission chains from sequencing and clinical data. Our TB-specific comparative evaluation of published algorithms (4–8) found low agreement between predicted and presumed source cases identified locally in an investigation of a large TB cluster with low genetic diversity. Furthermore, the variability between runs (except for SeqTrack) may make these algorithms impractical for use in public health decision-making during active investigations because differing outputs could be difficult to reconcile for use in guiding public health interventions. In contrast, LITT outputs were highly concordant with source cases identified locally during an investigation, with LITT and the investigation agreeing on the same most likely source 80% of the time; many of the discrepancies were due to specific data issues. Taken together, LITT agreed with investigation presumed sources more than other methods. This difference most likely results from the fact that LITT is based on field investigation work and incorporates much more information, such as clinical data, infectious periods, and known epidemiologic links, than other algorithms. The other algorithms include only WGS and sample collection date with parameter estimates for timing of illness. Thus, LITT is a novel approach to automating transmission detection. The LITT algorithm provides a standardized method for integrating WGS, clinical and epidemiological data in order to infer directionality of *Mtb* transmission (i.e., chains of transmission) by generating a filtered and ranked list of potential sources for every given case in the cluster.

We validated the LITT algorithm retrospectively using data from 56 cluster investigations, which represented a diverse range of cluster sizes and transmission circumstances. These clusters come from three high burden U.S. jurisdictions with densely sampled sequencing results. Future follow-up work could include validating LITT's performance prospectively with larger sample sizes and other cluster or outbreak settings. Furthermore, although the investigation presumed sources were used as a gold standard, these determinations have some uncertainty and can be prone to human error (23). For example, 12 investigation presumed source cases were refuted by large SNP differences that became apparent after the investigation once WGS data were available. Also, some predictions could not be validated because the local investigation had not identified a source. These cases generally occurred earlier in the cluster and were not prioritized for investigation. However, the fact that LITT was able to identify a most likely source for 39% of cases that did not have an investigation presumed source allows investigators to consider additional potential transmission chains. There is no guarantee that the investigation presumed sources are the true source cases, just that they are the most likely source given available data. Some amount of imprecision may be acceptable, however, as a primary goal of investigation may be to make overall inferences about transmission at the cluster (not patient) level.

LITT is meant to facilitate the analysis of investigation data and cannot replace a thorough field investigation. One limitation of LITT is that it will not detect missing cases. If the true source for a given case is not in the dataset, LITT may incorrectly identify another case as a potential source. However, LITT does not assume that all cases in the input dataset are part of the same chain of transmission and does not require that all patients have a sequenced isolate. Therefore, it may predict multiple transmission chains or cases without any potential sources. These scenarios are indications that there may be additional cases that have yet to be identified. Likewise, LITT will not detect missing epidemiologic links, which is an important consideration because links were particularly discriminatory for the investigators when they identified presumed sources (i.e., almost all cases with a presumed source had at least one epidemiologic link). However, LITT can consider shared risk factors, which are user-defined and often reflect additional data from the investigation, and LITT can be run with and without epidemiologic links to compare how epidemiologic links that are currently known influence the putative transmission network. We have found that in clusters with relatively few or no known epidemiologic links and low SNP diversity, potential source rankings will be driven only by the estimates of disease timing and infectiousness of cases, resulting in predictions that are difficult to validate. Thus, epidemiologic relationships are a crucial part of the LITT analysis.

In fact, as with all algorithms, the completeness and accuracy of input data will determine LITT's performance in identifying a potential source case. For example, if an infectious period is miscalculated, which often happens due to recall bias from self-reported symptoms, the true source may be ruled out. However, due to LITT's rapid run-time, users can compare results with different infectious period lengths when there is uncertainty with an infectious period. Furthermore, because many of the discrepancies in our review were explained by data entry errors, the review highlighted an additional use of LITT as a tool for data quality control in situations where transmission chains are already well-understood. It can be challenging to maintain current data and presumed source determinations during an investigation (e.g., data are changing and staff have competing priorities). Thus, using LITT can assist the investigation by helping to identify issues with data quality, and by systematically updating predictions as data change. Although LITT may highlight situations when data are illogical, there is no substitute for data completeness and quality.

We designed LITT to incorporate WGS, clinical, and epidemiologic data that are commonly available at the national level by virtue of being included in the national case-based surveillance system for TB in the United States. However, local staff may have additional data systems or knowledge that refute LITT predictions. For example, five of LITT's most likely potential sources in the detailed review were refuted using TST conversion results. We did not include TST conversion data because it was not routinely available (i.e., typically only a single TST result is reported to the national surveillance system); future work may revisit this decision. Thus, we encourage LITT users to carefully review the full list of potential sources and make best use of additional data and local investigators' knowledge.

LITT scores provided strong discriminatory power between investigation presumed sources and other potential sources; scores can be categorized to help frontline public health staff interpret most likely sources in a structured framework. Furthermore, the required data inputs provide a guide for what data need to be collected and a useful structure for analyzing the data. Given the short runtime (<60 seconds on a laptop), LITT can increase efficiency and save data analysis time. We have made LITT accessible to users without R programming expertise by developing a user's manual, training resources, and an R Shiny graphical user interface that outputs Excel spreadsheets. Thus, LITT can help investigators quickly, systematically, and repeatedly analyze data to identify and rank potential source cases over the course of an investigation. The application of LITT, particularly for large clusters with complex data sources, can help public health staff use molecular and epidemiologic information together to prioritize strategies and resources that prevent TB disease and interrupt transmission.

## Data Availability Statement

The data analyzed in this study are subject to the following licenses/restrictions: Data were shared for the purposes of this project under a data use agreement and are only available via state and local public health department jurisdictions due to necessary patient privacy protections. Requests to access these datasets should be directed to Kathryn Winglee, nrf1@cdc.gov.

## Author Contributions

KW designed the algorithm, abstracted data, wrote the code for the algorithm, and authored the paper. CM, KR, and ST advised on algorithm design, abstracted data, and reviewed the paper. LL performed the detailed evaluation data review, advised on algorithm design, and reviewed the paper. SK and SR advised on algorithm design, provided statistical guidance, and reviewed the paper. MC, WN, JK, JS, SP, and TS assisted with and performed field investigations and data collection, advised on algorithm design, and reviewed the paper. LC, JP, and ST assisted with the collection and analysis of WGS data. BS advised on algorithm design, supervised the project, edited the paper, and obtained funding for the project. All authors contributed to the article and approved the submitted version.

## Conflict of Interest

The authors declare that the research was conducted in the absence of any commercial or financial relationships that could be construed as a potential conflict of interest.

## Abbreviations

CDPH: California Department of Public Health
CDC: Centers for Disease Control and Prevention
IA: infection acquisition
IP: infectious period
LA DPH: Los Angeles County Department of Public Health
LITT: Logically Inferred Tuberculosis Transmission
*Mtb*: *Mycobacterium tuberculosis*
NYC DOHMH: New York City Department of Health and Mental Hygiene
SCOTTI: Structured Coalescent Transmission Tree Inference
SNP: single nucleotide polymorphism
TB: tuberculosis
TST: tuberculin skin test
WGS: whole-genome sequencing.
