# Supplementary material for: Logically Inferred Tuberculosis Transmission (LITT): A Data Integration Algorithm to Rank Potential Source Cases
Source: Front Public Health. 2021 Jun 21;9:667337. doi: 10.3389/fpubh.2021.667337 (PMC8255782; doi:10.3389/fpubh.2021.667337)
Supplement: Supplementary file 1 [file Data_Sheet_1.pdf]

Supplementary Web Material

Logically Inferred Tuberculosis Transmission (LITT): A Data Integration Algorithm to Rank Potential Source Cases

Kathryn Winglee, Clinton J. McDaniel, Lauren Linde, Steve Kammerer, Martin Cilnis, Kala M. Raz, Wendy Noboa, Jillian Knorr, Lauren Cowan, Sue Reynolds, James Posey, Jeanne Sullivan Meissner, Shameer Poonja, Tambi Shaw, Sarah Talarico, Benjamin J. Silk

Contents

Appendix 1: Technical appendix for the Logically Inferred Tuberculosis Transmission (LITT) method to integrate *Mycobacterium tuberculosis* whole-genome sequencing, clinical, and epidemiological data to identify and rank potential tuberculosis source cases 3

1.1 Data compilation 3

1.2 Date calculations for LITT input 4

1.3 LITT algorithm filtering 5

1.4 LITT algorithm scoring 6

1.5 Hypothetical example 8

1.6 LITT score analysis 9

1.7 LITT app use 9

Appendix 2: An evaluation of five previously published algorithms for integrating *Mycobacterium tuberculosis* whole-genome sequencing, clinical, and epidemiological data to identify and rank potential tuberculosis source cases 11

2.1 Evaluation methods 11

2.2 Algorithm-specific configurations 11

2.3 Evaluation results 12

Supplementary Figures 13

Supplementary Figure 1. Frequency distributions of number of tuberculosis cases in each cluster (Figure 1A) and the proportions of cases in each cluster that had presumed source cases identified by an investigation (Figure 1B). 13

Supplementary Figure 2. Screenshot of the Logically Inferred Tuberculosis Transmission (LITT) user interface before uploading data. 14

Supplementary Figure 3. Comparison of the Logically Inferred Tuberculosis Transmission (LITT) algorithm's predicted source cases to presumed sources identified by local investigators by set. 16

Supplementary Figure 4. Frequency distributions of time and single nucleotide polymorphism (SNP) data for investigation given case-presumed source pairs in training set of tuberculosis cases. 17

Supplementary Figure 5. Frequency distribution of changes in date calculations when using precise input date data for estimating infectious period start date (e.g., symptom onset from clinical records) instead of surveillance data only. 19

Supplementary Tables 20

Supplementary Table 1. Epidemiologic link definitions used for data abstraction for clusters used to evaluate the Logically Inferred Tuberculosis Transmission (LITT) algorithm. 20

Supplementary Table 2. Comparisons of algorithms' predicted source cases to presumed source cases identified locally by investigation of a large outbreak of tuberculosis cases with whole-genome sequencing data (WGS) (n=29 cases).. 21

Supplementary Table 3. Numbers of all investigation given case-presumed source pairs in the training set by strength of presumed source determination and number of single nucleotide polymorphisms (SNPs), timing, epidemiologic linkage, and clinical evidence of infectiousness. 22

References 23

Appendix 1: Technical appendix for the Logically Inferred Tuberculosis Transmission (LITT) method to integrate *Mycobacterium tuberculosis* whole-genome sequencing, clinical, and epidemiological data to identify and rank potential tuberculosis source cases

This technical appendix describes how we evaluated LITT using retrospective data collected for diverse *M. tuberculosis* transmission circumstances in multiple high-burden locations in the United States (section 1.1). Furthermore, details for date calculation inputs (1.2), LITT filtering (1.3) and scoring (1.4), a hypothetical example (1.5), score analysis (1.6), and LITT app use (1.7) are described.

1.1 Data compilation

We used available data from tuberculosis (TB) cluster or outbreak investigations from three jurisdictions in the United States: the California Department of Public Health (CDPH), the Los Angeles County Department of Public Health (LA DPH), and the New York City Department of Health and Mental Hygiene (NYC DOHMH). In total, there were 56 clusters (cluster size range: 2–69 cases); all clusters had at least one source case identified by investigators (Table 2, Supplementary Figure 1), which was defined as the presumed source. CDPH investigates larger, more complex clusters in the state of California, and provided data on three large clusters that were representative of the kinds of large outbreaks that they see. LA DPH provided data on all 49 clusters with a presumed source that were investigated between January 2015 and November 2017. NYC DOHMH provided data on four large clusters that represented some of the different transmission scenarios that they have encountered. Thus, the LA DPH data provided a collection of cases across all genotype-matched clusters that occurred in a high burden county; the CDPH and NYC DOHMH data provided cases from large, complex outbreaks, including both community and location-based transmission.

Cases were included in a cluster if: i) they had matching genotyping results using the combination of 24-locus MIRU-VNTR (mycobacterial interspersed repetitive units variable number of tandem repeats) and spoligotype (spacer oligonucleotide typing), or ii) a closely related genotype (1-2 MIRU-VNTR loci different), or iii) no genotyping data were available, but they had an epidemiologic relationship to a genotyped case in a cluster. Whole-genome sequencing (WGS) was performed for select *M. tuberculosis* isolates as part of the investigation and whole-genome single nucleotide polymorphism (SNP) comparison and phylogenetic analysis was performed for all sequenced isolates in a cluster using BioNumerics 7.6.3 (Applied Maths, Sint-Martens-Latem, Belgium). Briefly, sequence reads were aligned to the reference genome *M. tuberculosis* H37Rv (NC_000962.3) and SNPs were filtered to produce a list of high-quality, informative SNPs for each cluster using the Strict SNP filtering (Closed SNP set) SNP analysis template within the BioNumerics 7.6.3 software. The identified high-quality, informative SNPs were then used to construct a phylogenetic tree and pairwise SNP distance matrix using the neighbor-joining method. Some cases were not included in the phylogenetic analysis because the case was culture-negative, the isolate was no longer available for sequencing, the isolate was contaminated, or the isolate had a low depth of sequencing coverage. All clusters included in the analysis had at least two *M. tuberculosis* isolates with phylogenetic analysis results from WGS data; however, 32/56 (57%) of our clusters were missing WGS data on at least one case.

We manually reviewed investigation records for each cluster. As available, we also abstracted symptom onset date, infectious period, any known epidemiologic linkages, and any presumed sources identified for each case.

These data originated from investigation activities, which included patient interviews, contact investigations, chart abstractions, and record searches. Investigators categorized epidemiologic links into three different strengths, which varied between clusters (see Supplementary Table 1 for explanations of the most commonly used epidemiologic link strength definitions). All clusters had at least one known epidemiologic link, although epidemiologic links are not required to run LITT. Presumed sources, which were identified by investigators based on their general experience and specific knowledge of case timing, infectiousness, and epidemiologic relationships between cases, were used as the gold standard for evaluations. If WGS data were available, it also went into the presumed source determinations. However, WGS did not become available for most LA DPH clusters until after the investigations (i.e., WGS was not part of these presumed source determinations). CDPH subjectively categorized their presumed source determinations into definite (i.e., investigators were confident a case was the presumed source), probable (i.e., investigators were somewhat less confident a case was the presumed source), and possible (i.e., investigators thought a case was the most likely source, but were not confident in this determination). NYC DOHMH and LAC DPH did not classify their presumed sources, so all presumed sources were classified as definite. In our evaluation, we treated all presumed sources equally for simplicity despite varying levels of confidence. These investigation data were then combined with routine surveillance data (1), which contained additional data on timing (e.g., sample collection date and treatment start date) as well as clinical data, including the site of disease, age, and sputum smear microscopy result and cavitary status on chest radiograph.

For development of the LITT algorithm, we divided our 56 clusters into training and testing datasets. The training dataset used to develop the algorithm consisted of the three clusters from CDPH and 27 clusters from LA DPH (n=293 TB cases). This data was used to develop filtering and scoring criteria and cutoffs. The testing dataset contained the remaining 22 clusters from LA DPH and the 4 clusters from NYC DOHMH (n=241 TB cases) and was used to assess LITT's performance.

1.2 Date calculations for LITT input

Infectious periods: For adult cases with pulmonary or laryngeal TB, we calculated infectious period (IP) start by first calculating the earliest date that the patient had TB disease documented, based on surveillance data and any investigation data if available. This earliest date is the first of: the date the first isolate was collected for which drug susceptibility testing was done, treatment start date, TB case count date, TB case report date, specimen collection date, or symptom onset date. If we had an IP start from the investigation but no symptom onset, symptom onset date was calculated as 3 months after the investigation IP start and then input into the earliest date calculation. The IP start was then calculated as three months before the earliest date; if the investigation had calculated an IP start, we used that date if it was at least one month before the earliest date (since symptoms are not part of the dataset, this accommodates the recommendation for patients with no symptoms, are sputum smear negative and have no cavitary chest radiograph). These determinations are based on U.S. Centers for Disease Control and Prevention (CDC) guidelines (2).

For adult cases with pulmonary or laryngeal TB, we used the IP end date from the investigation, if the local program had calculated this date. Otherwise, we calculated IP end as 2 weeks after treatment start date if treatment start date was available. If no treatment start date or investigation IP end date were available and the patient had died, we used the date therapy stopped; if this date was not available, we used date of death, or report date if death date was also not available. If none of these dates were available, the IP end was not calculated. If no treatment start date or investigation IP end date were available and the patient had completed treatment, we used two weeks after the latest of the same dates used for the IP start date calculation described above. These calculations are also based on CDC guidelines (2).

Infection acquisition periods: In addition to the IP, LITT will analyze an infection acquisition (IA) period to account for the timing of when a patient could have been infected. The IA start date represents the earliest date that a case could have been infected. In our analyses, it was calculated using the approximate date of birth for pediatric cases (cases < 10 years old) or the date of arrival for non-U.S.–born cases. The IA end date represents the latest date that a case could have been infected, allowing time for the patient to develop symptoms. It is only used for extrapulmonary and pediatric cases (i.e., patients that cannot be a source). The IA end date is calculated the same as the IP start date; however, it is analyzed as a separate variable because exclusively extrapulmonary (not including laryngeal TB) and pediatric TB cases are generally not infectious.

1.3 LITT algorithm filtering

Cases appropriately included in a cluster are defined by the user; all cases with an IP start or IA end date in the inputs will be included in the analysis. LITT begins by selecting one case as the given case, the case for which it is trying to identify potential source cases. For each given case, LITT begins with all other cases in the cluster as the set of potential sources. Then, based on four criteria, LITT filters (removes) from that set any case that could not be a source for the given case:

1. **Genetic distance**: if both the given case and potential source have sequenced isolates, the potential source is filtered if the *M. tuberculosis* isolates are >5 SNPs from one another. This default cutoff of 5 SNPs is based on current CDC experiences and an analysis of our given case-presumed source pairs. However, the LITT user can change this default as needed. If one or both cases do not have sequencing data (i.e., there is no SNP distance data for the pair), the potential source is filtered unless it has an epidemiologic link or shared risk factor with the given case.

    Note: Appendix Figure 4 shows the frequency distributions of SNP distances between all presumed transmission pairs (Supplementary Figure 4C) and transmission pairs ascertained independent of WGS (i.e., sequencing analyzed after investigation) (Supplementary Figure 4D). Nearly all isolates for definite, probable, or possible presumed source cases are within 8 SNPs of the given cases' isolates. In the training set, the majority are within 5 SNPs (Supplementary Table 3). The larger SNP distances in Supplementary Figure 4C and 4D (SNP distances) indicate the presumed source is unlikely to be the true source and are examples of situations where investigators did not update predictions when new data became available (if sequencing was available during the investigation), thus highlighting the role for LITT in maintaining data quality by helping to systematically update predictions.

2. **Disease site**: all potential sources must have an infectious form of TB disease (i.e., pulmonary or laryngeal TB). Any noninfectious case will be filtered from the set of potential sources. Any case missing this data will default to assuming an infectious form of TB disease.

3. **Sequential timing**: potential source cases are filtered if they are not infectious until after the given case has acquired TB (i.e., LITT filters a potential source if the IP start date is after the given case's IP start date). However, LITT allows buffer time for imprecision in estimating IP start, which is dependent on certainty in date calculations. If the investigation did not identify symptom onset date or calculate an IP start, then the case is classified as only having less precise surveillance data; otherwise, the case is considered to have more precise date data (i.e., symptom onset dates or other clinical data not derived from a surveillance system). For pediatric and extrapulmonary cases, LITT uses the IA end date instead of IP start date; here, we refer to the IP start date throughout for simplicity.

a. If both the given case and potential source have precise (e.g., symptom onset) date data, then LITT allows a buffer of 3 months (i.e., potential source cases whose IP start date is >3 months after the given case's IP start are filtered).
b. If either the given case or the potential source case has imprecise (surveillance) date data only, then LITT allows a buffer of 5 months.
c. If both cases have imprecise date data, then LITT allows a buffer of 6 months.

Note: These buffers are based on a comparison of how date data change the estimates of the earliest date a patient had TB. Using the training dataset, Supplementary Figure 5 shows a change of 2.2 months earlier on average in estimating infectious period start date with precise input date data; we rounded up to 3 months to be conservative.

In addition, if IA start is available for a given case, LITT filters any potential sources whose IP end (or IP start if IP end is not available) is before the IA start, as these cases stopped being infectious before the given case could have been infected.

4. **Age**: all potential sources must be 10 years or older (2); any case less than 10 years old will be filtered from the set of potential sources. Cases missing age data will be assumed to be adult by default.

1.4 LITT algorithm scoring

After applying the four filtering criteria, LITT evaluates the remaining potential source cases using a scoring system. (If no potential source cases remain, LITT moves to the next given case; outputs indicate that the given case did not have any potential sources in the cluster.) The score is set up *inversely*; the lower the value, the more likely that case is the source for the given case. A total score is calculated by adding together values from four scoring variables (Table 1).

1. **Genetic distance between *M. tuberculosis* isolates (SNP rating)**: the number of SNPs between isolates from the potential source and given case is used by LITT as a rating score to measure how closely the isolates are related genetically (i.e., a small or no SNP difference suggests that case is more likely to be a potential source); this distance does not account for phylogenetic tree structure, which users should review visually. This variable is left blank if SNP distance for the pair is not available.

2. **Patient infectiousness (infectiousness rating)**: patients with a cavitary form of disease identified on chest radiograph are generally most infectious (LITT rating score=0) regardless of sputum smear microscopy results; without cavitary disease, patients with positive sputum smear results (score=1) are typically more infectious than patients with smear negativity (2). Patients with smear negative and non-cavitary findings can transmit *M. tuberculosis* but are generally least likely to transmit *M. tuberculosis* (3, 4), so they are assigned a high rating (score=5). Cases missing cavitary status data will be assumed not to have cavitary disease; cases that are missing smear status data will be assumed to be smear negative.

   Note: In the LITT training dataset, 84 (97%) of 87 definite given case-presumed source pairs had a cavitary form of disease identified on chest radiograph or positive sputum smear microscopy (Supplementary Table 3). Among all 115 given case-presumed source pairs (i.e., definite, probable, or possible), 54 (47%) had cavitary disease and 58 (50%) were sputum smear positive without cavitary disease.

3. **Timing of cases (disease timing rating)**: if the potential source's IP start is after the given case's IP start due to the time buffer period (see 1.3 above), LITT gives the potential source a higher rating (LITT rating score=2) for sequential timing (i.e., less likely source). If the potential source's IP end date is two or more years before the given case's IP start date, the potential source is given a lower rating (score=1). Otherwise, the potential source is given a lowest rating score of 0, indicating a likely source based on timing.

   Note: These ratings are based in part on data from published studies, which have suggested that most patients develop disease within two years of infection (5, 6). This finding is consistent with the LITT training dataset, which shows the distributions of time intervals from presumed source case IP start date (Supplementary Figure 4A) or IP end dates (Supplementary Figure 4B) to given case IP start dates. Among all 115 given case-presumed source pairs (i.e., definite, probable, or possible), 95 (83%) of the sources had IP end dates less than two years before the given case's IP end date (including sources with an IP start date after the given case IP start date) (Supplementary Table 3).

4. **Epidemiologic relationship between cases (epidemiologic and shared risk factor rating)**: if an epidemiologic link has been identified between a potential source and given case, LITT uses the strength of the link as a rating score (i.e., a stronger link is a lower score, indicating that case is more likely to be the source). If multiple links are identified between the potential source and given case pair, the strongest link strength is used. If no strength data input is provided, then a default, intermediate strength of probable is used (LITT rating score=1). If no epidemiologic link has been identified, LITT will look for shared risk factors. These risk factors are optional variables that are specifically relevant to the transmission within the cluster (e.g., sheltered patients experiencing homelessness). Multiple risk factors can be assigned by investigators. If there are multiple risk factors, risk factors can be weighted by relative importance to transmission. LITT will ensure the relative risk factor weights sum to 1 (range: 0 to 1). If the given case and potential source do not share an epidemiologic link but risk factors have been provided for the cluster, then the epidemiologic and risk factor rating starts with a highest rating score of 3 (i.e., no epidemiologic link). For each risk factor that the given case and the potential source share, the weight of that risk factor is subtracted from the rating score. Thus, if the cases share all risk factors, then a rating score of 2 will be applied (i.e., equal to a possible epidemiologic link); if some are shared, the rating score will be between 2 and 3; if none are shared (or no risk factors are provided), the rating score will be 3.

   Note: In the LITT training dataset, 84 (97%) of 87 definite presumed source cases had a definite or probable epidemiologic link to the given case (Supplementary Table 3). Among all 115 given case-presumed source case pairs (i.e., definite, probable, or possible), 73 (63%) had definite and 26 (23%) had probable epidemiologic links.

Total scoring, without-SNP scoring, and additional filtering: Once score ratings are completed for each possible source case, LITT calculates a total score by adding together the SNP rating, infectiousness rating, disease timing rating, and epidemiologic and shared risk factor rating. The total score is designated as missing by LITT for those given case-potential source pairs that do not have a SNP distance (i.e., WGS data are missing). In addition, LITT calculates a without-SNP score by adding together the infectiousness rating, disease timing rating, and epidemiologic and shared risk factor ratings only. Once the scores have been calculated, LITT performs a final filter to remove potential sources with a total score of 8 or more or a without-SNP score of 5 or more (if a given case-potential source pair does not have a SNP distance). This final filter helps limit the number of potential sources, so that investigators can focus on the most likely potential sources. The remaining potential sources are categorized into high, medium, and low likelihood based on their score. The final filtering score cutoffs (8 for total

score and 5 for without-SNP score) are based on the median score values for the potential sources that were not presumed sources from the investigation (see Figure 3A). Additional details are described in the LITT score analysis below (section 1.6).

Ranking: LITT then ranks the remaining potential sources for line list output. First, LITT sorts in ascending order all potential sources by total score (i.e., cases have WGS data to derive a SNP distance to the given case); case(s) with the lowest score are given a rank of 1 as the most likely potential source(s). Two or more potential sources with the same score are given the same rank; rank order below the tied scores then skips down to the next case.

If not all cases have a total score (i.e., some are missing WGS and thus the SNP rating), LITT then sorts those potential sources by the without-SNP score in ascending order. If there is a mix of potential source cases with total scores and without-SNP scores, LITT merges the two lists by inserting the potential sources that do not have WGS results as a tie with the first potential source that has WGS and that also has the same or higher without-SNP score. The total score and without-SNP score for sequenced cases are not always in the same order (e.g., a relatively large SNP distance could make a case with a low without-SNP score have a high total score), so the LITT output indicates that the non-sequenced cases inserted into the ranked list were evaluated differently. Investigators are encouraged to consider non-sequenced cases, especially since they must share an epidemiologic link or risk factors with the given case to pass LITT filter criteria.

1.5 Hypothetical example

Consider the cluster of 10 cases shown in Figure 1. LITT starts with one of the cases as the given case (central white circle). The algorithm then considers the remaining nine cases (cases A–I, black circles). First, LITT filters out cases that could not be the source for the given case (see Appendix 1.3). Case C was filtered due to genetic distance (i.e., there were more than five SNPs between Case C's isolate and the given case's isolate). Case E was filtered based on disease site (i.e., this patient did not have pulmonary or laryngeal TB). Case F was filtered due to timing (i.e., the IP start was after the given case's IP start, suggesting that Case E became infectious after the given case was already infected). Case H was filtered due to young age (i.e., patients less than ten years old generally are not infectious).

The remaining five potential source cases are then scored by LITT (see Appendix 1.4 and Table 1). Cases A, B, D, G, and I each had pulmonary TB, occurred in an adult patient, and were neither genetically distant nor temporally too late (i.e., they were not filtered). Case D was ranked first and had the lowest possible score (0), so this case had a high likelihood of being the presumed source for the given case. Case D's score was determined by the combination of four criteria: having an *M. tuberculosis* isolate that was zero SNPs from the given case's isolate (0), sputum smear positivity and cavitary disease on chest radiograph indicating a higher degree of infectiousness (0), the patient's infectious period ending 1 year before the given case's infectious period start (0), and a definite epidemiologic link to the given case (0) (i.e., score = 0 + 0 + 0 + 0 = 0).

Cases B and G were tied as ranked second. Their scores were determined by: having an isolate that was 1 SNP from the given case's isolate (1), sputum smear positivity and cavitary disease on chest radiograph indicating a higher degree of infectiousness (0), infectious periods ending more than 2 years before the given case's infectious period start (1), and a definite epidemiologic link to the given case (0). Thus, both cases had a score of 2 (1 + 0 + 1 + 0). Since both B and G had the same score, they are given the same rank (i.e., they are tied); since a score of 2 is the second highest score among the set of potential sources for the given case, they are both ranked second. A score of two indicates B and G have a medium likelihood of being the source for the given case.

The score for Case A was determined by: having an isolate that was 1 SNP from the given case's isolate (1), sputum smear positivity, but no cavitary disease on chest radiograph indicating lower infectiousness (1), an infectious period ending more than 2 years before the given case's infectious period start (1), and a probable epidemiologic

link to the given case (1). Thus, Case A had a score of 4 (1 + 1 + 1 + 1), which has a low likelihood of being the source for the given case. Case A is given a rank of 4 (i.e., Cases D, B, and G are more likely potential source cases for the given case). Note the rank of 3 was skipped because B and G had the same score and so both ranked second.

The score for Case I was determined by: having an isolate that was zero SNPs from the given case's isolate (0), sputum smear negativity and no cavitary disease on chest radiograph indicating little evidence of infectiousness (5), an infectious period starting 1 month after the given case's infectious period start (i.e., in the buffer period) (2), and a possible epidemiologic link to the given case (2). Thus, Case I had a score of 9 (0 + 5 + 2 + 2). Case I is filtered from the final list of potential sources, as it is very unlikely to be the source for the given case.

After this analysis, the user could conclude that Case D is the most likely source for the given case based on current data. However, Cases B, G, and A are all potential sources that should also be considered.

LITT will then repeat this entire evaluation process with each next case in the cluster (e.g., Case A) as the given case until every case in the cluster has been designated and evaluated as the given case.

1.6 LITT score analysis

To make the LITT scores more interpretable to frontline TB control program staff, we established cutoffs for categorizing potential sources' scores as high, medium, and low likelihood. We analyzed the LITT total scores and without-SNP scores for all unfiltered given case-potential source pairs. For each set of scores with a whole number value (i.e., no risk factors were used in the analysis), we tabulated the proportions of pairs identified as presumed sources by the local investigation out of the total numbers of given case-potential source pairs with each score. We calculated 95% confidence intervals using the Pearson-Klopper (exact) method for binomial counts, using the binom.confint function of the R binom package (version 1.1-1) (7). P-values were calculated using a Wilcoxon rank sum test. These proportions increased with lower scores (Figure 3B), indicating that the scores estimate the likelihood that a given case-potential source case pair would be chosen as the presumed source by the investigation. To facilitate use, we divided the range of scores into four different likelihood categories. We used a proportion of 0.75 as a cutoff for high likelihood (total score of 0 or 1, with or without SNPs), a proportion between 0.25 and 0.75 for medium likelihood (total score of 3 or 4, or without-SNP score of 2), and a proportion below 0.25 for low likelihood (total score > 4, or without-SNP score > 3). Finally, we used the median of the other potential sources (8 for total score and 5 for without-SNP score) as a cutoff to filter pairs from consideration, as they are very unlikely to be sources for the given case (Figure 3A).

1.7 LITT app use

To make LITT more accessible to frontline staff, LITT is available as an R Shiny app (8) (Supplementary Figure 2). The app's online user interface expects LITT users to input data from four Microsoft Excel tables:

(i) A case data table, which contains clinical characteristics of each case (status of sputum smear and cavitation on chest radiograph), the sequential timing of each case (IPs and IAs, as applicable), and any risk factors. All cases listed in this table will be treated as a given case by LITT;

(ii) An epidemiologic link table, which contains data on the epidemiologic relationships between cases;

(iii) A SNP matrix, which contains the number of SNPs between every isolate pair of cases with sequencing; and

(iv) A table of risk factor weights, if the user chooses to include risk factors in the analysis.

Once the user runs the program, LITT will generate a ranked list of potential sources for each case. The output is a set of Excel files that allow the user to review the potential sources for each given case, or the reason a potential source was filtered. Output files include a heatmap to give a quick visual summary, and node and link lists for upload into network visualization tools, such as MicrobeTrace (9). A LITT user's manual, training (mock) datasets, training presentation, input file templates, and all code written in R (10) are available at: https://github.com/CDCgov/TB_molecular_epidemiology.

Appendix 2: An evaluation of five previously published algorithms for integrating *Mycobacterium tuberculosis* whole-genome sequencing, clinical, and epidemiological data to identify and rank potential tuberculosis source cases

This technical appendix describes our evaluation methods (section 2.1), including specific configurations (2.2), and results (2.3) in applying algorithms that use whole-genome sequencing (WGS) data to infer transmission directionality for a tuberculosis (TB) outbreak.

2.1 Evaluation methods

In August of 2017, we reviewed published literature and identified five algorithms designed to infer pathogen transmission chains from WGS data that had a publicly available implementation (e.g., code available to run in an R package): SeqTrack (11), outbreaker (12), Structured Coalescent Transmission Tree Inference (SCOTTI) (13), TransPhylo (14), and phybreak(15).

We selected our large outbreak of 69 tuberculosis (TB) cases to evaluate the five different algorithms for *M. tuberculosis* transmission. Given that most algorithms can only infer events for cases with sequenced isolates, we subset the data to include only cases with WGS data available as of September 2017 (i.e., when the analysis was first run), leaving us with 48 cases, some of which were distantly related (> 5 SNPs). Initial attempts at analysis with all 48 cases resulted in computationally long run times and many predictions of unsampled cases (SCOTTI and TransPhylo can predict whether there are missing cases in the transmission chain, which could occur for many reasons, including the case not having WGS results or not yet being detected). As a result, we further subset the cluster data to only include 29 cases with *M. tuberculosis* isolates that were closely related genetically and centered around a case with multiple known transmission events. We ran SeqTrack, outbreaker, SCOTTI, TransPhylo, phybreak, and LITT on these 29 cases for comparison purposes.

Of these 29 cases, 19 (66%) had a presumed source identified by the local investigation. To evaluate the algorithms' results, the most likely source predicted for each of these 19 cases was compared to this presumed source. Each algorithm was run three times on the same input data to account for results that might not remain consistent between runs due to the random sampling used in several algorithms. The number of times the algorithm and local investigation agreed on the most likely source was averaged across the three runs. All analyses were performed in R (10).

2.2 Algorithm-specific configurations

For the comparative evaluation, each algorithm was specifically configured as follows (with the seed set to 0 for all analyses):

- We ran SeqTrack (11) three times in R using version 2.1.1 of the adegenet package with the SNP distance matrix and sample collection dates for the 29 isolates.

- We ran Outbreaker version 1.1-8 (12) three times using the SNP distance matrix and sample collection dates for the 29 isolates. For the generation time distribution, we used the discr_si function in the EpiEstim R package(16) (version 2.2-1) to calculate a discretized shifted gamma distribution with a mean of 1,338.3 days and standard deviation of 115.1 days. These numbers come from the distribution of SNPs distances divided by 0.5 (i.e., mutation rate estimated by Walker et al. (17)), then multiplied by 365

to convert to days. We used the get.tTree function to generate the transmission network used for comparison to the investigation presumed source cases.

- We ran SCOTTI (13) version 2.0.1 in BEAST 2.5.1 (18) three times on the whole-genome sequences aligned to *Mycobacterium tuberculosis* H37Rv of 29 isolates. First, BEAST 2.5.1 was run, using the default settings in the SCOTTI_generate_xml.py script, except that the infectious period was used for the dates, the maximum number of hosts was set to 65 (i.e., reflecting the number of cases in the cluster including non-sequenced isolates and cases identified after the date cutoff) and 10^5 iterations were used. We used SCOTTI's Make_transmission_tree_alternative.py script to generate the transmission network for comparison to the investigation presumed source cases. The most likely direct source predicted by SCOTTI was unsampled for all cases; for comparison purposes, the most likely sampled case was used instead.

- For TransPhylo (14), we used BEAST (19) version 1.8.4 to generate a timed phylogeny from the whole-genome sequences of the 29 isolates; we used default settings with a general time reversible model and a strict molecular clock. BEAST was run for 10^7 iterations with the parameter state recorded every 1,000 iterations and the first 10% discarded as burn-in. We then ran TransPhylo version 1.2.3 three times using the BEAST timed phylogeny as input, the same distributions as described by Didelot et al. (14) (i.e., Gamma distribution for the sampling time density with a shape parameter 1.1 and rate 0.4, Gamma distribution for generation time density with shape parameter 1.3 and rate parameter 0.3, with 10^5 iterations) and a starting sampling probability of 0.9. We used the consTTree function to generate the transmission network for comparison to the presumed source cases identified by local investigation.

- We ran Phybreak (15) version 0.2.0 three times using whole-genome sequences aligned to *Mycobacterium tuberculosis* H37Rv and sample collection dates of the 29 isolates. Default values were used, except for the parameters for which inference for *M. tuberculosis* was provided by Klinkenberg et al. (i.e., mutation rate μ of 3.4×10^{-10}, mean generation interval m_G of 107, mean sampling interval m_s of 419, and within-host slope r of 0.88)(15). Phybreak identified 30 (1 run) or 31 (2 runs) unsampled cases. For comparison purposes, if the source of a sampled case was an unsampled case, the first sampled ancestral infector of that unsampled case was used instead of the unsampled case.

- We ran LITT three times on the 29 isolates using the SNP matrix and investigation data (epidemiologic links and infectious periods) as described above for the 29 isolates with the default 5 SNP cutoff.

2.3 Evaluation results

All algorithms evaluated except LITT had low proportions of source case agreement with the presumed source cases identified by local investigation (Supplementary Table 2). However, if a probability was estimated, all algorithms evaluated had low probabilities for every source identified in their predictions. Except for SeqTrack and LITT, all algorithms produced most likely transmission chains that differed between runs. These algorithms use Bayesian approaches to sample from a distribution, and all had low support for any one source (<0.5 and often <0.2). We suspect the variation between runs despite setting the seed is due to the fact that minor differences when sampling were enough to change the relative ranking of potential source cases, given low overall support and random sampling. Most likely sources produced by SCOTTI had the most agreement (18%) with the presumed source cases identified by investigation. In contrast, LITT produced the same most likely source case in every run, and correctly identified 17 (89%) of the 19 presumed source cases as the most likely source case.

Supplementary Figures

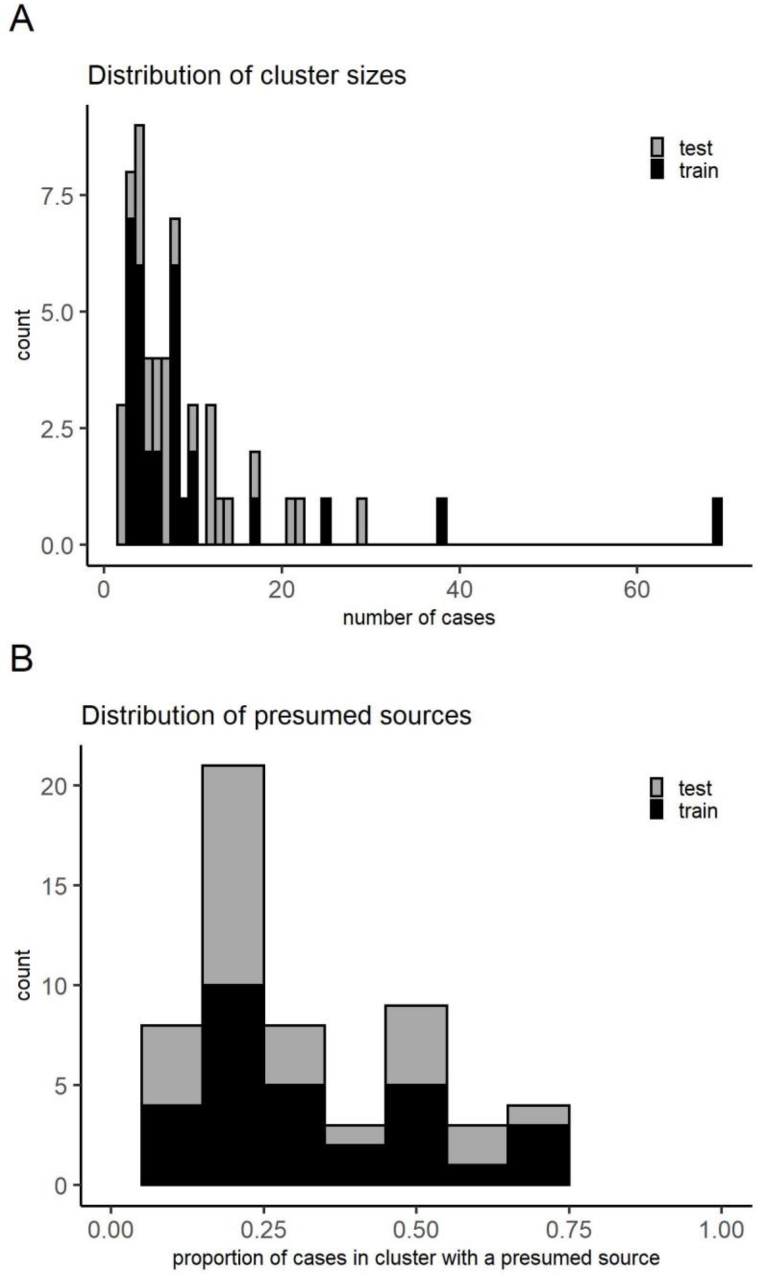

Supplementary Figure 1. Frequency distributions of number of tuberculosis cases in each cluster (Figure 1A) and the proportions of cases in each cluster that had presumed source cases identified by an investigation (Figure 1B).

Bars are colored for clusters used to train (black) or test (grey) the Logically Inferred Tuberculosis Transmission (LITT) algorithm. The training dataset consisted of the three clusters from California Department of Public Health and 27 clusters from Los Angeles County Department of Public Health; the test dataset contained the remaining 22 clusters from Los Angeles County Department of Public Health and the 4 clusters from New York City Department of Health and Mental Hygiene (see data compilation, section 1.1).

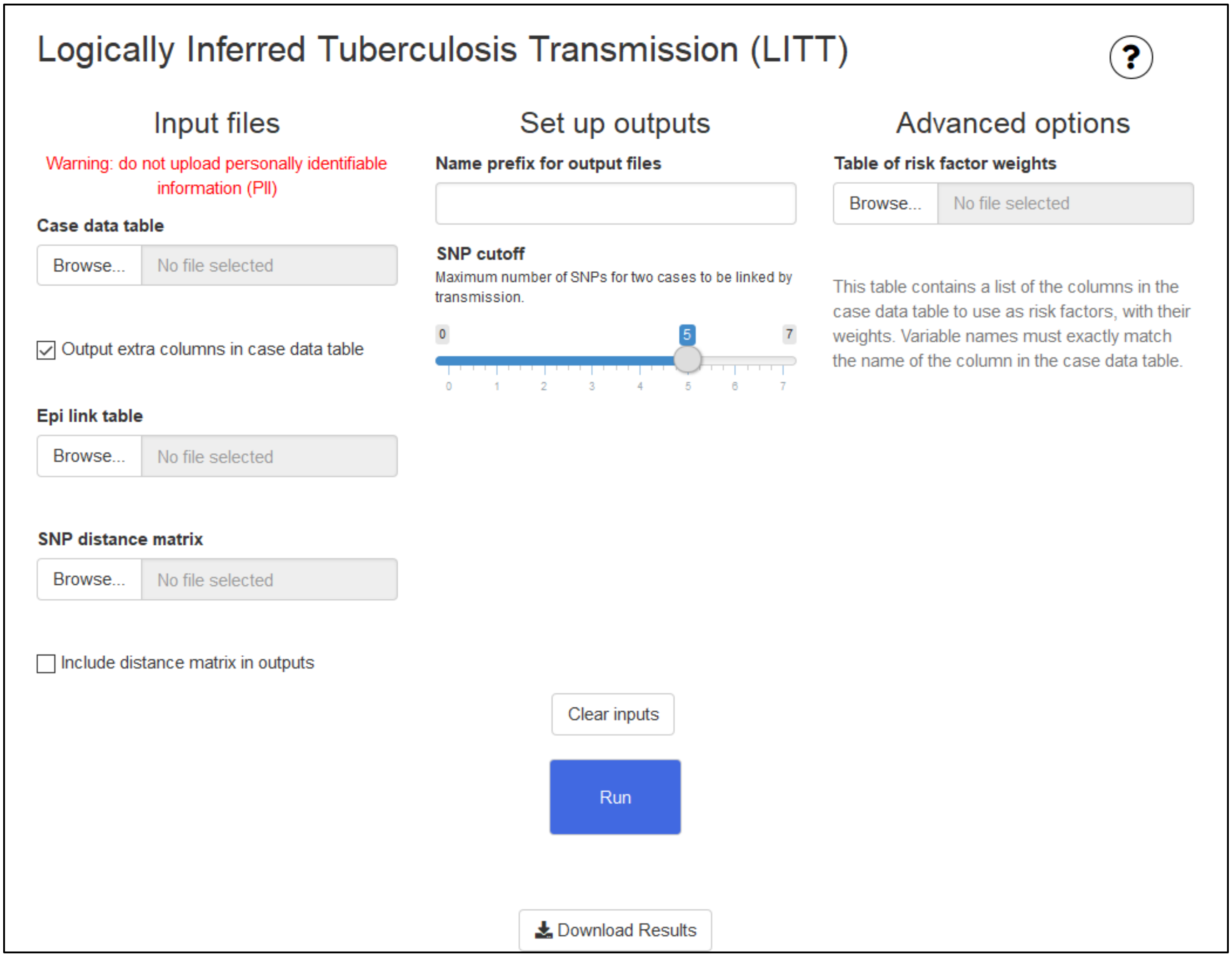

Supplementary Figure 2. Screenshot of the Logically Inferred Tuberculosis Transmission (LITT) user interface before uploading data.

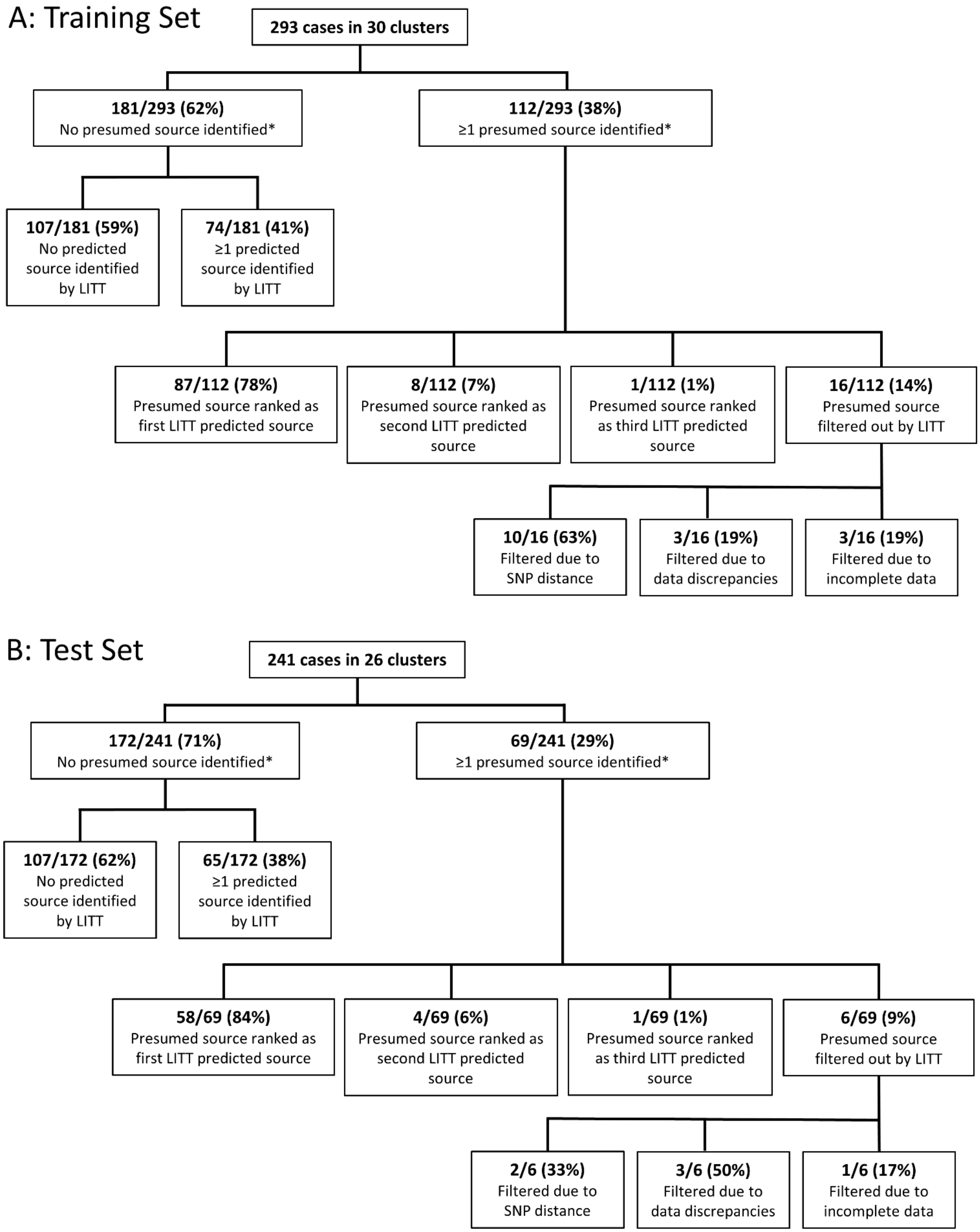
A: Training Set
293 cases in 30 clusters
181/293 (62%)
No presumed source identified*
112/293 (38%)
≥1 presumed source identified*
107/181 (59%)
No predicted source identified by LITT
74/181 (41%)
≥1 predicted source identified by LITT
87/112 (78%)
Presumed source ranked as first LITT predicted source
8/112 (7%)
Presumed source ranked as second LITT predicted source
1/112 (1%)
Presumed source ranked as third LITT predicted source
16/112 (14%)
Presumed source filtered out by LITT
10/16 (63%)
Filtered due to SNP distance
3/16 (19%)
Filtered due to data discrepancies
3/16 (19%)
Filtered due to incomplete data
B: Test Set
241 cases in 26 clusters
172/241 (71%)
No presumed source identified*
69/241 (29%)
≥1 presumed source identified*
107/172 (62%)
No predicted source identified by LITT
65/172 (38%)
≥1 predicted source identified by LITT
58/69 (84%)
Presumed source ranked as first LITT predicted source
4/69 (6%)
Presumed source ranked as second LITT predicted source
1/69 (1%)
Presumed source ranked as third LITT predicted source
6/69 (9%)
Presumed source filtered out by LITT
2/6 (33%)
Filtered due to SNP distance
3/6 (50%)
Filtered due to data discrepancies
1/6 (17%)
Filtered due to incomplete data

Supplementary Figure 3. Comparison of the Logically Inferred Tuberculosis Transmission (LITT) algorithm's predicted source cases to presumed sources identified by local investigators by set.
(**A**) Clusters used to train the LITT algorithm.

(**B**) Clusters used to test the LITT algorithm.

* Presumed source cases of *Mycobacterium tuberculosis* transmission identified by public health investigations conducted by the California Department of Public Health, Los Angeles County Department of Public Health, New York City Department of Health and Mental Hygiene, and their partners.

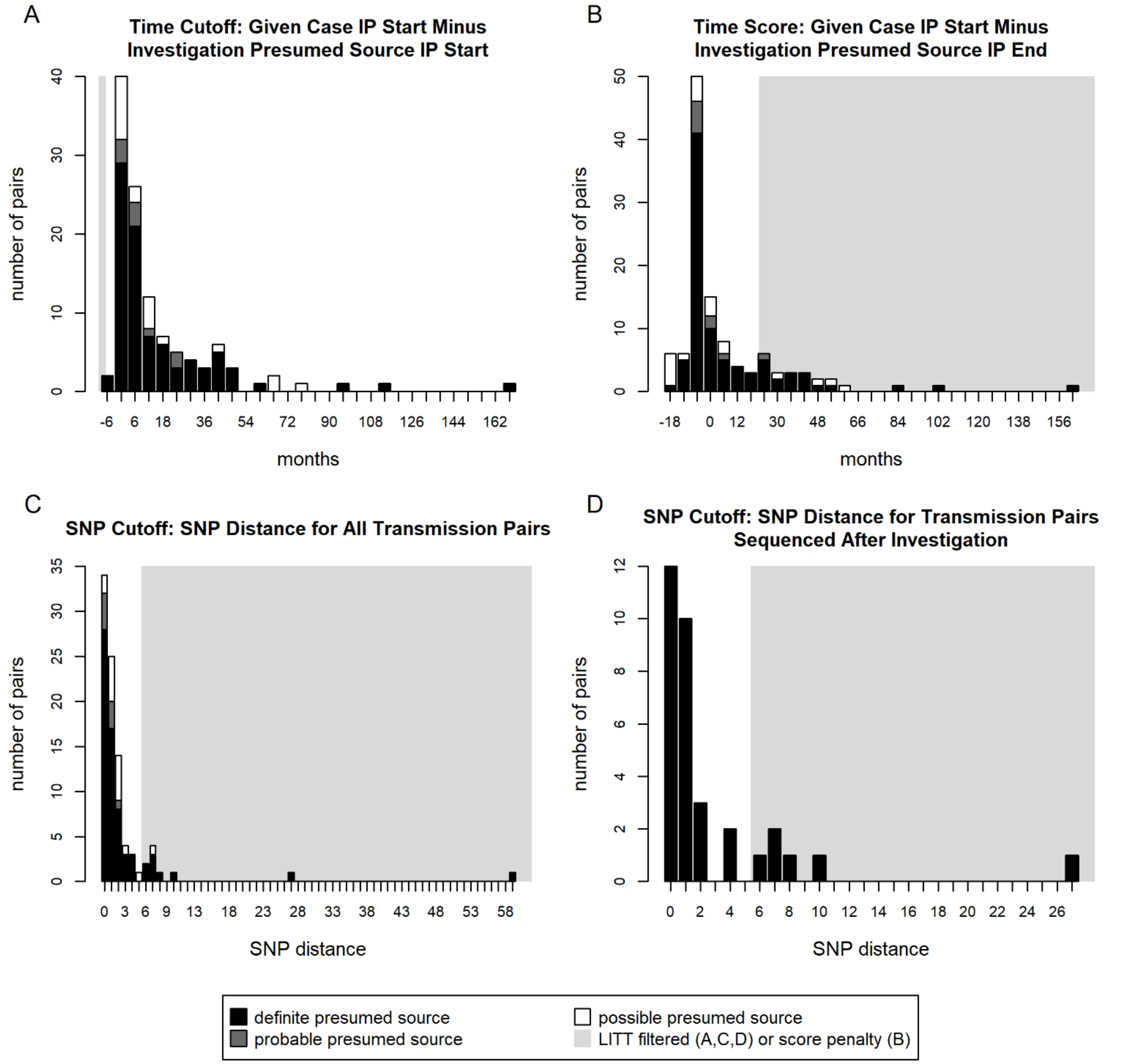

Supplementary Figure 4. Frequency distributions of time and single nucleotide polymorphism (SNP) data for investigation given case-presumed source pairs in training set of tuberculosis cases.
The training dataset for the Logically Inferred Tuberculosis Transmission (LITT) algorithm consisted of the three clusters from California Department of Public Health and 27 clusters from Los Angeles County Department of Public Health (see data compilation, section 1.1). In all figures, black indicates a definite presumed source, dark gray indicates a probable presumed source, and white indicates a possible presumed source (i.e., investigation was confident a case was the presumed source, somewhat less confident, or investigation thought a case was the most likely source, but were not confident in this determination, respectively). Abbreviations**:** SNP = single nucleotide polymorphism; IP = infectious period.

(**A**) Time difference (in months) between source IP start dates of the given case and investigation presumed case, which is used to filter potential sources (N=115 pairs). A negative number indicates that the investigation

presumed source IP start was after the given case IP start. The light gray shaded area indicates the pairs that would be filtered because they do not meet the 6-month (maximum) time buffer cutoff.

(**B**) Time difference (in months) between given case IP start date and investigation presumed source IP end date, which is used to evaluate and score the sequential timing of pairs (N=115 pairs). The light gray shaded area indicates the pairs where the given case IP start is more than 2 years after the presumed source IP end; these pairs would receive the sequential timing rating score of 1 (see table 1 for scoring system).

(**C**) SNP distance between all sequenced pairs of given cases and investigation presumed sources (N=91 pairs). The light gray shaded area indicates pairs that would be filtered because they have a genetic distance >5 SNPs.

(**D**) SNP distance between sequenced pairs of given cases and investigation presumed sources, after removing pairs that had sequencing data delivered at the time of the investigation (N=33 pairs). Removing given cases and investigation presumed sources pairs with sequencing data delivered at the time of the investigation could prevent bias, as the number of SNPs is considered by investigators when making a presumed source determination.

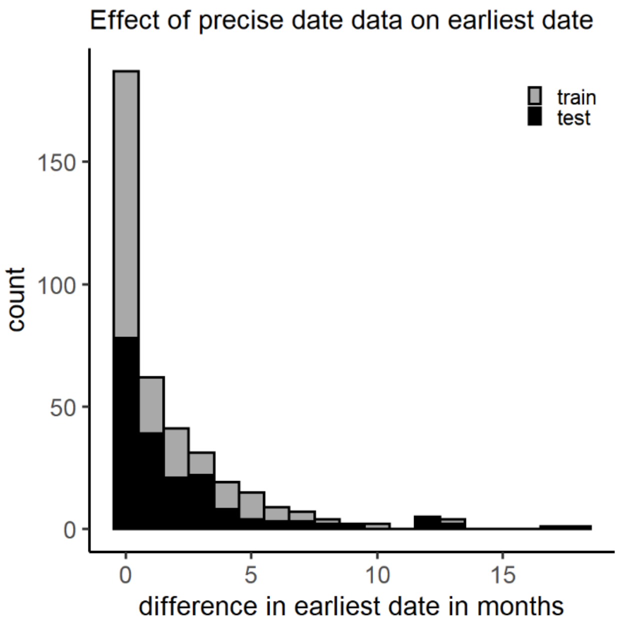

Supplementary Figure 5. Frequency distribution of changes in date calculations when using precise input date data for estimating infectious period start date (e.g., symptom onset from clinical records) instead of surveillance data only.

Bars are colored for cases used to train (black; N=200) or test (grey; N=190) the Logically Inferred Tuberculosis Transmission (LITT) algorithm.

Supplementary Tables

Supplementary Table 1. Epidemiologic link definitions used for data abstraction for clusters used to evaluate the Logically Inferred Tuberculosis Transmission (LITT) algorithm.

Epidemiologic link strength	Definition of close-association link	Definition of location-based link
Definite	Two patients named the other as a contact during an interview (either or both)	Two patients were confirmed (e.g., using an electronic registry) to have shared airspace at the same location (e.g., workplace, shelter) at the same time and during one case's infectious period
Probable	Two patients had a named contact in common, but did not directly name each other as contacts, OR<br><br>A person-search database, social media site, or internet search indicates that the patients are related, associated, or have shared a home address, OR<br><br>Third party reports two patients had close contact with one another	Two patients shared airspace at the same location during the same general time period, but unclear whether they were at the same location during one case's infectious period
Possible	Two patients have a matching last name, but no additional information is available to link patients	Patients share social or behavioral risk factors (e.g., substance use, homelessness, history of incarceration) that increase the risk of *M. tuberculosis* transmission, but dates and locations are unspecified, OR<br><br>Two patients reside in the same neighborhood

Supplementary Table 2. Comparisons of algorithms' predicted source cases to presumed source cases identified locally by investigation of a large outbreak of tuberculosis cases with whole-genome sequencing data (WGS) (n=29 cases).

Algorithm	**Average number (percent) times most likely predicted source matched presumed source in three runs*** **	**Number (percent) of cases with the same source in all three runs******	**Number (percent) of cases with the same source in two of the three runs******	**Number (percent) of cases with a different source in all three runs******	**Reference**
SeqTrack	3.0 (15.8%)	29 (100.0%)	0 (0.0%)	0 (0.0%)	(11)
outbreaker	0.3 (1.8%)	5 (17.2%)	5 (17.2%)	19 (65.5%)	(12)
SCOTTI*	3.3 (17.5%)	27 (93.1%)	2 (6.9%)	0 (0.0%)	(13)
TransPhylo*	2.0 (10.5%)	13 (44.8%)	6 (20.7%)	10 (34.5%)	(14)
phybreak	2.3 (12.3%)	0 (0.0%)	7 (24.1%)	22 (75.9%)	(15)
LITT**	17.0 (89.5%)	29 (100.0%)	0 (0.0%)	0 (0.0%)	

* SCOTTI and TransPhylo both identified unsampled cases as source cases; for comparison purposes, if the most likely source was an unsampled case, the sampled case that infected the unsampled case was used as the most likely source.

** Logically Inferred Tuberculosis Transmission (LITT) is a novel method to integrate WGS with clinical and epidemiological data to identify and rank potential TB source cases.

*** Average number (percent) based on the 19 TB cases with WGS data that had a presumed source identified by the local investigation.

**** Average number (percent) reflect all 29 cases with WGS data that were analyzed.

Supplementary Table 3. Numbers of all investigation given case-presumed source pairs in the training set by strength of presumed source determination and number of single nucleotide polymorphisms (SNPs), timing, epidemiologic linkage, and clinical evidence of infectiousness.

	**Presumed Source Determination***		
	Definite	Probable	Possible
**Number of SNPs between isolates**			
0 SNPs	28	4	2
1 SNPs	17	3	5
2 SNPs	8	1	5
3 SNPs	3	0	1
4 SNPs	3	0	0
5 SNPs	0	0	1
>5 SNPs	9	0	1
No SNP data	19	1	4
**Sequential timing**			
Source IP start date is before given case IP start date and source IP end < 2 years before given case IP start date	62	7	8
Source IP end date ≥ 2 years before given case IP start date	16	0	4
Source IP start date after given case IP start date	9	2	7
**Clinical evidence of infectiousness**			
Cavitary disease on chest radiograph**	37	2	15
No cavitary disease, positive sputum smear microscopy	47	7	4
No cavitary disease, negative sputum smear microscopy	3	0	0
**Strength of epidemiologic link**			
Definite epidemiologic link	71	2	0
Probable epidemiologic link	13	7	6
Possible epidemiologic link	3	0	11
No epidemiologic link	0	0	2

* Presumed source cases of *Mycobacterium tuberculosis* transmission were identified by public health investigations conducted by the California Department of Public Health (CDPH), Los Angeles Department of Public Health (LA DPH), and their partners. This data represents our training set of three clusters from CDPH and 27 clusters from LA DPH and includes all presumed sources for a given case, even if multiple presumed sources were identified or later review refuted them.

** Source case has cavitary disease on chest radiograph regardless of sputum smear microscopy results.

References

1. Ghosh S, Moonan PK, Cowan L, Grant J, Kammerer S, Navin TR. Tuberculosis genotyping information management system: enhancing tuberculosis surveillance in the United States. Infect Genet Evol. 2012;12(4):782-8.
2. National Tuberculosis Controllers Association, Centers for Disease Control and Prevention. Guidelines for the investigation of contacts of persons with infectious tuberculosis: Recommendations from the National Tuberculosis Controllers Association and CDC. MMWR Recomm Rep. 2005;54(RR-15):1-47.
3. Hernandez-Garduno E, Cook V, Kunimoto D, Elwood RK, Black WA, FitzGerald JM. Transmission of tuberculosis from smear negative patients: a molecular epidemiology study. Thorax. 2004;59(4):286-90.
4. Tostmann A, Kik SV, Kalisvaart NA, Sebek MM, Verver S, Boeree MJ, et al. Tuberculosis transmission by patients with smear-negative pulmonary tuberculosis in a large cohort in the Netherlands. Clin Infect Dis. 2008;47(9):1135-42.
5. Behr MA, Edelstein PH, Ramakrishnan L. Revisiting the timetable of tuberculosis. BMJ. 2018;362:k2738.
6. Reichler MR, Khan A, Sterling TR, Zhao H, Moran J, McAuley J, et al. Risk and Timing of Tuberculosis Among Close Contacts of Persons with Infectious Tuberculosis. J Infect Dis. 2018;218(6):1000-8.
7. Dorai-Raj S. binom: Binomial Confidence Intervals For Several Parameterizations. R package version 1.1-1 ed2014.
8. Chang W, Cheng J, Allaire J, Xie Y, McPherson J. shiny: Web Application Framework for R. 2019.
9. Campbell EM, Boyles A, Shankar A, Kim J, Knyazev S, Switzer WM. MicrobeTrace: Retooling Molecular Epidemiology for Rapid Public Health Response. bioRxiv. 2020;2020.07.22.216275.
10. R Core Team. R: A language and environment for statistical computing. Vienna, Austria: R Foundation for Statistical Computing; 2019.
11. Jombart T, Eggo RM, Dodd PJ, Balloux F. Reconstructing disease outbreaks from genetic data: a graph approach. Heredity (Edinb). 2011;106(2):383-90.
12. Jombart T, Cori A, Didelot X, Cauchemez S, Fraser C, Ferguson N. Bayesian reconstruction of disease outbreaks by combining epidemiologic and genomic data. PLoS Comput Biol. 2014;10(1):e1003457.
13. De Maio N, Wu CH, Wilson DJ. SCOTTI: Efficient Reconstruction of Transmission within Outbreaks with the Structured Coalescent. PLoS Comput Biol. 2016;12(9):e1005130.
14. Didelot X, Fraser C, Gardy J, Colijn C. Genomic Infectious Disease Epidemiology in Partially Sampled and Ongoing Outbreaks. Mol Biol Evol. 2017;34(4):997-1007.
15. Klinkenberg D, Backer JA, Didelot X, Colijn C, Wallinga J. Simultaneous inference of phylogenetic and transmission trees in infectious disease outbreaks. PLoS Comput Biol. 2017;13(5):e1005495.
16. Cori A, Ferguson NM, Fraser C, Cauchemez S. A new framework and software to estimate time-varying reproduction numbers during epidemics. Am J Epidemiol. 2013;178(9):1505-12.
17. Walker TM, Ip CL, Harrell RH, Evans JT, Kapatai G, Dedicoat MJ, et al. Whole-genome sequencing to delineate Mycobacterium tuberculosis outbreaks: a retrospective observational study. Lancet Infect Dis. 2013;13(2):137-46.
18. Bouckaert R, Vaughan TG, Barido-Sottani J, Duchene S, Fourment M, Gavryushkina A, et al. BEAST 2.5: An advanced software platform for Bayesian evolutionary analysis. PLoS Comput Biol. 2019;15(4):e1006650.
19. Drummond AJ, Suchard MA, Xie D, Rambaut A. Bayesian phylogenetics with BEAUti and the BEAST 1.7. Mol Biol Evol. 2012;29(8):1969-73.
